# 78,000-year-old record of Middle and Later Stone Age innovation in an East African tropical forest

**DOI:** 10.1038/s41467-018-04057-3

**Published:** 2018-05-09

**Authors:** Ceri Shipton, Patrick Roberts, Will Archer, Simon J. Armitage, Caesar Bita, James Blinkhorn, Colin Courtney-Mustaphi, Alison Crowther, Richard Curtis, Francesco d’ Errico, Katerina Douka, Patrick Faulkner, Huw S. Groucutt, Richard Helm, Andy I. R Herries, Severinus Jembe, Nikos Kourampas, Julia Lee-Thorp, Rob Marchant, Julio Mercader, Africa Pitarch Marti, Mary E. Prendergast, Ben Rowson, Amini Tengeza, Ruth Tibesasa, Tom S. White, Michael D. Petraglia, Nicole Boivin

**Affiliations:** 1 McDonald Institute for Archaeological Research, University of Cambridge, Downing Street Cambridge, Cambridge, CB2 3DZ UK; 2 British Institute in Eastern Africa, Laikipia Road, Kileleshwa, Nairobi Kenya; 3 Centre of Excellence for Australian Biodiversity and Heritage, Australian National University, Canberra, ACT 2601 Australia; 4 Department of Archaeology, Max Planck Institute for the Science of Human History, Kahlaische Strasse 10, Jena, D-07745 Germany; 5 Department of Human Evolution, Max Planck Institute for Evolutionary Anthropology, Deutscher Pl. 6, Leipzig, 04103 Germany; 6 Department of Archaeology, University of Cape Town, Rondebosch, 7701 Western Cape South Africa; 7 Department of Geography, Royal Holloway, University of London, Egham, Surrey TW20 OEX UK; 8 SSF Centre for Early Sapiens Behavior (SapienCe), University of Bergen, Øysteinsgate 3, Postboks 7805, Bergen, 5020 Norway; 9 Malindi Museum, National Museums of Kenya, Malindi, Kenya; 10 Department of Archaeology, Classics and Egyptology, University of Liverpool, 12–14 Abercromby Square, Liverpool, L69 7WZ UK; 11 Department Environment, York Institute for Tropical Ecosystems, University of York, Heslington, York, YO10 5NG UK; 12 School of Social Sciences, The University of Queensland, St Lucia, QLD 4072 Australia; 13 Department of Archaeology and History, The Australian Archaeomagnetism Laboratory, Palaeoscience Labs, La Trobe University, Melbourne Campus, Bundoora, VIC 3086 Australia; 14 UMR 5199 PACEA, CNRS/Université de Bordeaux, Bâtiment B18, Allée Geoffroy Saint Hilaire, CS, 50023 - 33615 PESSAC CEDEX, France; 15 Research Laboratory for Archaeology and the History of Art, Dyson Perrins Building, South Parks Road, Oxford, OX1 3QY UK; 16 Faculty of Arts and Social Sciences, Department of Archaeology, The University of Sydney, Sydney, NSW Australia; 17 School of Archaeology, University of Oxford, 36 Beaumont Street, Oxford, OX1 2PG UK; 18 Canterbury Archaeological Trust, 92A Broad Street, Canterbury, Kent CT1 2LU UK; 19 Coastal Forests Conservation Unit, National Museums of Kenya, Kilifi, Kenya; 20 Centre for Open Learning, University of Edinburgh, Paterson’s Land, Edinburgh, EH8 8AQ Scotland UK; 21 Biological and Environmental Sciences, University of Stirling, Stirling, FK9 4LA Scotland UK; 22 Department of Anthropology and Archaeology, University of Calgary, 2500 University Drive, Calgary, AB T2N 1N4 Canada; 23 Grup de Recerca Aplicada al Patrimoni Cultural (GRAPAC), Department of Animal Biology, Plant Biology and Ecology, Faculty of Biosciences, Autonomous University of Barcelona (UAB), Campus Bellaterra, Bellaterra, 08193 Spain; 24 Department of Sociology and Anthropology, Saint Louis University, Avenida del Valle 34, Madrid, 28003 Spain; 25 Invertebrate Biodiversity, National Museum Wales, Cathays Park, Cardiff, CF10 3NP UK; 26 Department of Anthropology and Archaeology, University of Pretoria, cnr Lynnwood Road and Roper Street, Hatfield, South Africa; 27 University Museum of Zoology, Cambridge Downing Street, Cambridge, CB2 3EJ UK; 28 Department of Life Sciences, The Natural History Museum, Cromwell Road, London, SW7 5BD UK; 29 Human Origins Program, Smithsonian Institution, Washington, DC 20560 USA

## Abstract

The Middle to Later Stone Age transition in Africa has been debated as a significant shift in human technological, cultural, and cognitive evolution. However, the majority of research on this transition is currently focused on southern Africa due to a lack of long-term, stratified sites across much of the African continent. Here, we report a 78,000-year-long archeological record from Panga ya Saidi, a cave in the humid coastal forest of Kenya. Following a shift in toolkits ~67,000 years ago, novel symbolic and technological behaviors assemble in a non-unilinear manner. Against a backdrop of a persistent tropical forest-grassland ecotone, localized innovations better characterize the Late Pleistocene of this part of East Africa than alternative emphases on dramatic revolutions or migrations.

## Abstract (web summary)

Most of the archaeological record of the Middle to Later Stone Age transition comes from southern Africa. Here, Shipton et al. describe the new site Panga ya Saidi on the coast of Kenya that covers the last 78,000 years and shows gradual cultural and technological change in the Late Pleistocene.

## Introduction

The terms Middle Stone Age (MSA) and Later Stone Age (LSA) have long been used to frame discussions of behavioral and cultural change in Africa^1^. Changes in lithic production (such as making elongate blades and stone-tipped arrows^2, 3^), the appearance of symbolic material culture^4, 5^, and subsistence diversification^3^ associated with the MSA and LSA have all been identified as important thresholds in human cognitive and social evolution^3, 6^. Many researchers have highlighted the revolutionary nature of MSA and LSA human capacities, in some cases arguing that they reflect cognitive evolutionary developments^7^ or that they stimulated pan-African and global migrations from 60,000 years ago (ka) onwards^8, 9^. On the other hand, recent discoveries in southern Africa have suggested a more gradual development of these material culture traits^10–13^.

The debate as to the significance and tempo of behavioral changes during the MSA and LSA has largely focussed on the temperate and coastal environments of southern Africa^12, 14^. This is due to a general lack of well-dated, well-stratified records in other key areas of the African continent, particularly across the period 80–40 ka, though the Haua Fteah and Taforalt in North Africa are notable exceptions^15, 16^. Long-term, dated records from East Africa remain scarce. Several East African sites have produced evidence for novel practices, reflected in the appearance of backed stone tools and beads over the last 60–40,000 years^17–22^. However, the chronologies and environmental contexts of these key behavioral transitions are not clear. Sites >50,000 years old have only just begun to be identified beyond the East Africa Rift System^23^ and are limited to the Lake Victoria region^24–26^.

**Fig. 1:**
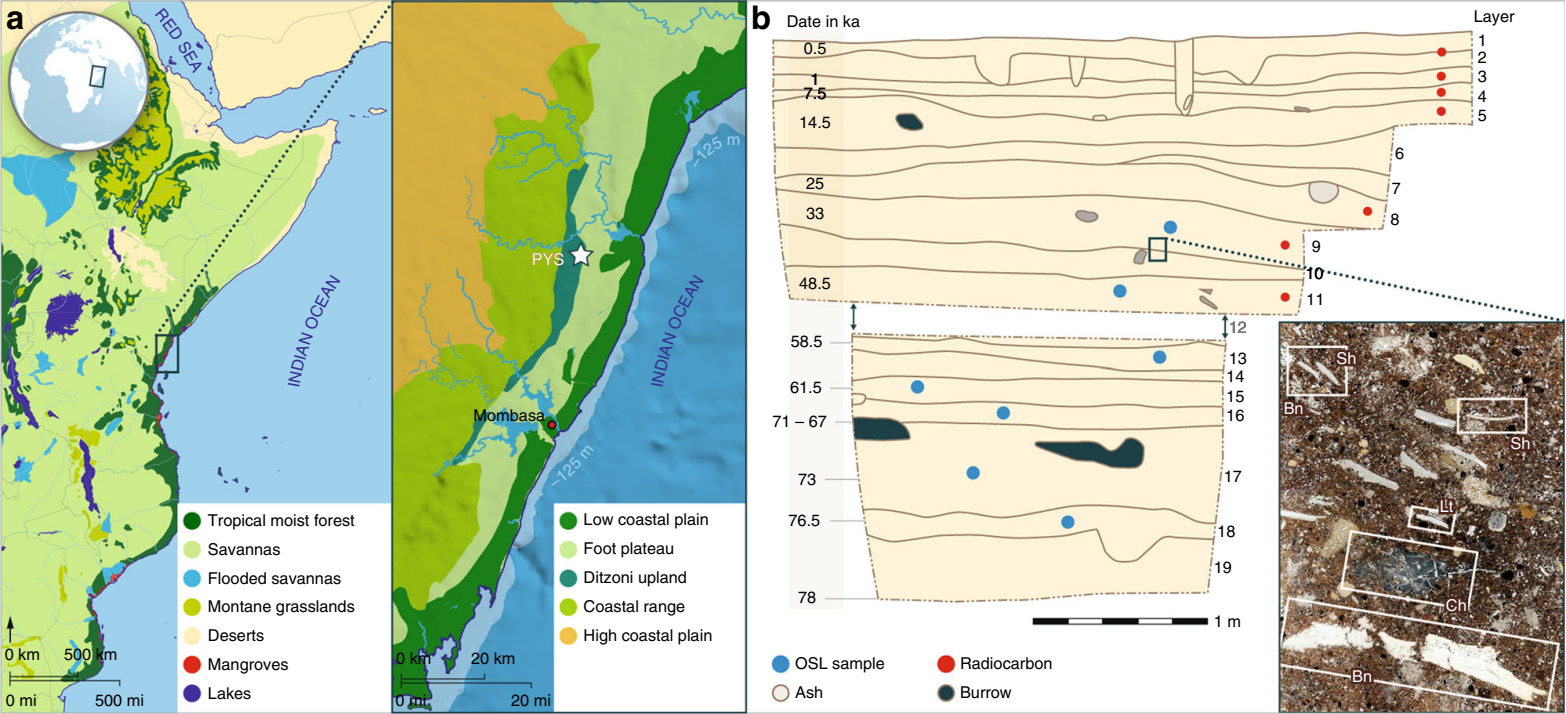
Environmental setting and PYS stratigraphic section. **a** The location of PYS in the tropical moist forest of coastal East Africa, situated in the Ditzoni upland, southeastern Kenya. **b** The stratigraphic sequence of PYS showing the Layers and modeled ages, with example of a micromorphological thin section, illustrating the rich biogenic/anthropogenic contents in the sediments (Sh shell, Bn bone, Lt lithic, Ch charcoal). Note that Layer 12 was not continuous across the whole excavation and did not occur in this section. Age estimates are shown as the median of the highest posterior density age range for simplicity

The new archeological cave site of Panga ya Saidi (PYS) described here offers an opportunity to address geographical and ecological biases in our understanding of early human behavioral and cultural change. The site is situated 15 km from the present-day shoreline in the Zanzibar-Inhambane coastal forest mosaic that runs along the East African littoral, with the coastal shelf dropping below −125 m depth within 5 km of the modern coastline (Fig. 1a) (Supplementary Note 1). The cave is in the Dzitsoni limestone hills on an ecotone between lowland tropical forest and savannah (SM5). Excavations at PYS have revealed exceptional preservation and stratigraphic integrity, and a record of human activity back to ~78 ka, including a rich technological sequence that includes lithic forms elsewhere associated with the MSA and LSA. Alongside a rich range of paleoecological indicators, these features mean that the site offers a rare opportunity to study human behavioral changes in an evolutionarily critical, but poorly-understood, region of Africa.

## Results

### Stratigraphy and chronology

The 3 m deep excavated sequence at PYS encompasses 19 layers (Supplementary Note 2) (Fig. 1b). Three lithological boundaries divide the profile into four main lithostratigraphic units that are discussed in detail in Supplementary Note 1. A series of 20 stratigraphically ordered and internally consistent radiocarbon and optically stimulated luminescence (OSL) age estimates, when included in a Bayesian model, show human occupation in every Marine Isotope Stage (MIS), from late MIS5 c. 78 ka into the Holocene (Supplementary Note 3). Geoarchaeological and micromorphological studies indicate that the sequence consists of fine colluvia, spalling, and anthropogenic deposits with abundant organic and cultural microremains (e.g., fauna, flora, lithic microdebitage – Supplementary Notes 4 and 5).

**Fig. 2:**
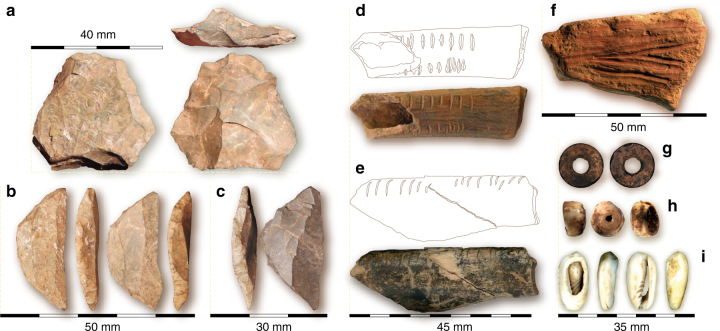
Selected artifacts from PYS. **a** Levallois core from Layer 11. **b** Two backed lithic artifacts from Layer 11. **c** Backed lithic artifact from Layer 3. **d** Notched bone from Layer 8. **e** Notched bone from Layer 9. **f** Ocher crayon from Layer 10. **g** Ostrich eggshell bead from Layer 8. **h** *Conus* shell bead from Layer 16. **i** Gastropod shell bead from Layer 4

Geomorphological and magnetic susceptibility proxies for human occupation intensity (Fig. 2; Supplementary Note 2) indicate a pattern of intermittent pulses of human activity. In the early part of the sequence, in Layers 19, 18, and the lower part of Layer 17 (~78–73 ka), occupation intensity is low. This is followed by a possible hiatus when both lithics and charcoal >c. 125 μm drop off, and magnetic susceptibility and biogenic input signals are dramatically reduced (Fig. 2). Human occupation proxies subsequently show a general trend of increasing intensity from Layer 16 (beginning ~67 ka) to the Holocene (Fig. 2).

### Cultural artifacts

**Fig. 3:**
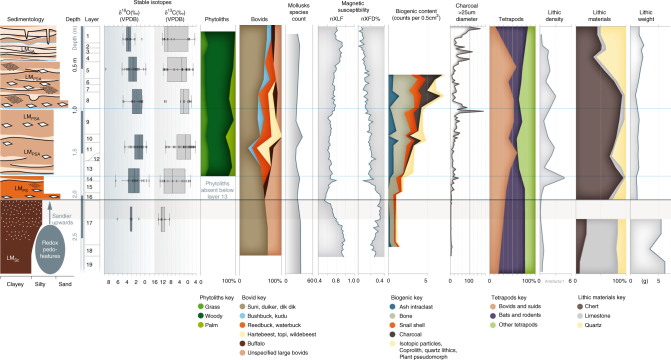
The palaeoenvironmental and human occupation proxies from PYS. From left to right: Sedimentology (LM(SC) sandy clayey loam, LM(PS) pebbly sandy loam, LM(PSA) pebbly sandy ashy loam, LM(SA) sandy ashy loam); Depth; Layer divisions; Box and whisker plots of stable oxygen and carbon isotope values of mammalian teeth; Phytoliths, showing the proportion of grass, palm, and woody taxa; Percentage of different bovids in Minimum Number of Individuals (MNI); Terrestrial mollusk rarefied species count; Magnetic susceptibility (XLF and XFD%); Biogenic content of micromorphology thin sections; Microcharcoal abundance; Proportions of selected faunal groups as a percentage of total tetrapod MNI; Lithic density; Lithic material types; Lithic weight (mean debitage weight)

The long artifactual sequence is comprised of 17 ocher fragments, eight worked bone artifacts, 88 ostrich eggshell beads, 27 marine shell beads, five exotic manuports, as well as >30,000 knapped stone artifacts, including Levallois cores and backed artifacts—typical of MSA and LSA technologies, respectively^6, 27, 28^ (Fig. 3). The metrics and characteristics of these technologies are discussed in greater detail in Supplementary Note 4. The possible hiatus or ephemeral occupation in the PYS sequence between 73 and 67 ka corresponds with a change in the stone artifact sequence. In the early part of the sequence stone artifacts are characterized by large flakes (Fig. 2), often made using variations of the Levallois method, and large retouched points (Supplementary Note 4), comfortably fitting with other contemporary MSA assemblages^29^. Immediately after the hiatus, in Layer 16, there is a shift in rock type proportions from principally microcrystalline limestone to cryptocrystalline quartz and chert, and a concomitant reduction in artifact size (Fig. 2, Supplementary Note 4). Size reduction is evident across all stone artifacts, including within cryptocrystalline materials and retouched tools, demonstrating that this change does not simply reflect variation in procured raw material package size (Supplementary Note 4).

Coupled with the switch to small cryptocrystalline stone tools after ~67 ka is an increased use of bipolar technology (Supplementary Fig. 4). A shift in technological emphasis toward bipolar strategies and a reduction in stone tool size is considered a key marker of LSA behavior elsewhere in Africa^27, 30, 31^. A change from Levallois to prismatic blade technology and the appearance of backed crescentic tools have also been highlighted as indicators of the LSA^6, 32^. However, in the PYS sequence, Levallois technology occurs alongside backed crescents in Layers 11 and 12 (~51–48 ka) and Layers 5–3 (~14–1 ka) (Supplementary Note 4) (Fig. 3). Prismatic blade production is rare in the PYS sequence, but bipolar and Levallois blades become common in the upper part of the sequence (Layers 8–3) (~25–1 ka) (Supplementary Note 4). Levallois and bipolar technology, blade production, and backed crescentic tools occur recurrently and intermittently, with no evidence for a unilinear accumulation of traits or a uniform uptake of the latter three traits as a package. Despite changes in technology, stone artifacts remain consistently small and were predominantly produced on cryptocrystalline materials after ~67 ka.

Beads, ocher fragments, and worked bone have been associated with behavioral complexity in the Late Pleistocene and occur with increasing regularity through the sequence at PYS (Supplementary Note 4). The earliest bead, a *Conus* sp. shell spire, occurs in Layer 16 which dates from between ~67–63 ka (Fig. 3). At ~33 ka (Layer 9) the most common beads were *Conus* shell spires (*n* = 13) (Fig. 3). Recurrent engagement with coastal resources for symbolic use is behavioral, rather than geographic, given minimal changes in distance from the shoreline during the site’s occupation (Fig. 1a) (Supplementary Note 1). Ostrich eggshell beads at PYS reach their highest frequency ~25 ka (Layer 8, *n* = 70), while fully-manufactured beads made of marine shells are the dominant types during the Holocene (Layers 1–5) (Fig. 2). Layers 8-10 (~48–25 ka) produced two modified ocher fragments (Fig. 3), as well as carved bone and tusk artifacts, including a decorated bone tube (Fig. 3) and a small bone point ; artifact types that have been argued to be characteristic of the LSA^28^. In contrast to many revolutionary or unilinear interpretations of technological and cultural development, the PYS sequence reveals a pattern of intermittent presence of different technological traits and symbolic artifacts that have been associated with the MSA and LSA.

### Paleoecology

Numerous proxies point to broad perseverance of tropical forest and grassland environments throughout the sequence (Supplementary Note 5). This is indicated, for example, by the consistently high (> 25 species per sample) terrestrial mollusk diversity, with most of the mollusk species requiring humid shady conditions.

Sedimentology and magnetic susceptibility data indicates a shift to drier conditions following the early occupation in Layers 17–19 ~78–73 ka (Fig. 2) (see also Supplementary Note 5). This is supported, albeit with a lag, by the stable isotope data which shows higher stable carbon (δ^13^C) in Layers 10–12 relative to 13–16 and 17 (Supplementary Tables 12 and 13), indicative of an increase in the presence of C_4_ resources, most likely in the form of grassland, in the diets of fauna being exploited at the site in the region *c*. 48 ka. Visually, δ^18^O also tracks this trend but there is no statistically significant difference between Layer 17 and Layers 13–16 or Layers 10–12 (Supplementary Tables 14 and 15).

From ~67–48 ka, paleoenvironmental proxies at PYS show that the local environment underwent relatively little variation until the final occupation of the cave at ~0.5 ka (Fig. 2). The wide δ^13^C and δ^18^O ranges of fauna after Layer 17 (Fig. 2) (Supplementary Data 1) reflects the persistence of C_3_ and C_4_ vegetation, and a variety of water sources affected to differing extents by evapotranspiration in the vicinity of PYS; likely in the form of a forest-grassland ecotonal setting. A consistent ecotonal situation is supported by zooarchaeological data for the persistent presence of open and bush/forest adapted mammalian species, as well as phytolith datasets that document the continued occurrence of woody, grass, and palm phytoliths in the immediate site environment (Fig. 2).

## Discussion

The paleoecological datasets from PYS agree with other East African records that point to a period of low amplitude environmental change throughout much of MIS4-1, particularly from records nearer the coast where the maritime influence buffers against temperature extremes^33, 34^. The consistent presence of the forest-grassland ecotone throughout the last ~67,000 years accompanies evidence for increasing occupation intensity at PYS, perhaps suggesting a growing human presence in the region linked to the use of mosaic habitats. Moreover, alongside the magnetic susceptibility data for increased occupation intensity from 60 ka, this may imply, as has been suggested elsewhere, that the MSA–LSA transition of East Africa is a long-term pattern of change related to growing population densities^33, 35^.

Indeed, the PYS sequence does not document a radical change in technological or cultural behavior in East Africa *~*60–50 ka that might be suggestive of cognitive or technological “revolutions” or migrations^7, 8^. Instead, PYS documents a long-term assembly, and intermittent presence, of various innovative traits. In particular, there is no dramatic appearance of an LSA technological package and, instead, older MSA technological traits, such as Levallois cores, exist alongside the development of backed artifacts and blade production. The principal change in the sequence is the reduction in lithic size and the shift to cryptocrystalline materials ~67 ka.

From MIS6-3 *Homo sapiens* began to adapt to a diversity of coastal^13, 36^, tropical forest^37, 38^, and hyper-cold^39, 40^ environments across Africa and Eurasia. Humans appear to have adapted locally to these environments, gradually developing new symbolic forms, technological production strategies, and subsistence behaviors. It seems that the Middle and Late Pleistocene of Africa is best characterized by diverse *Homo sapiens* populations, adopting a range of survival strategies and new forms of social communication on an intermittent, *ad hoc* basis in different environmental and climatic contexts^41, 42^. It is this adaptive plasticity that truly defines the expansion and development of humans as a global species.

## Methods

### Excavation

Our excavation is in a large rockshelter in the first chamber of the collapsed-roof cave, near to the main entrance. Three seasons of excavation between 2010 and 2013 have exposed a 3 m deep sequence, with the trench measuring 3.5 × 2 m at the top stepping into 2 × 1 m at the base. The trenches were excavated using the single context method according to the Museum of London protocols with the addition of total excavated sediment volume recorded for each context. Any animal burrows were excavated by hand and their contents discarded. In situ excavated deposits were dry-sieved on site through a 5 mm mesh.

A program of bulk soil sampling for flotation (0.5 mm) and wet-sieving (1 mm) to recover archeobotanical, zooarcheological, and paleoenvironmental microremains was also implemented (see below). Where possible, a minimum sample volume of 60 litres per context was maintained. Smaller contexts (<60 L) were sampled in their entirety. Nineteen stratigraphic layers were identified in the section, each corresponding to a particular excavation context. A continuous column bulk sediment sample was taken for palaeoenvironmental analyses with 100 g samples taken at every 2 cm of depth. Deposits exposed in PYS Trench 4 (2013 excavation) were logged on-site following standard sedimentological procedures^43–45^. Sediment color, texture, composition, structures, postdepositional disturbance and the nature, and geometry of layer boundaries were recorded for each layer resolved by the excavators. Layers were grouped into higher-order, multilayer lithostratigraphic units, as shown in Supplementary Fig. 1.

### Micromorphology

A set of 23 undisturbed micromorphology sediment samples were collected from the excavated profile in clear polyurethane boxes. In view of the large dimensions and stratigraphic complexity of the excavated trench, sampling was at a reconnaissance scale, concentrating on layer boundaries and distinctive features. Sample boxes were labeled, photographed and plotted on the profile drawing before removal from the profile (Supplementary Fig. 1). Out of this sample set, 10 samples from the Pleistocene part of the profile were processed for micromorphological analysis at the Thin Section Micromorphology Laboratory, University of Stirling (sample code PYS; thin sections manufactured by George MacLeod). Samples were air-dried and impregnated with polyester (polylite) resin following standard procedures (http://www.thin.stir.ac.uk/). Ca. 30 μm thick, uncovered, large format thin sections (7.5 × 11 cm) were manufactured from the hardened impregnated blocks.

Thin sections were observed with a polarizing microscope at magnifications of ×12.5 to ×400, using plain polarized (PPL), cross-polarized (XPL), and oblique incident light (OIL). Relative abundance of sediment/soil components was estimated using standard semi-quantitative estimation charts^46, 47^. Key sediment constituents larger than ca. 50 μm (mineral grains; pedoclasts; biogenic particles, etc.) were point-counted using a 0.5 × 0.5 cm grid overlay printed on clear acetate. Point-counted biogenic particles included bone fragments (from small cave vertebrates and larger vertebrate fauna—the latter possibly including human prey); small vertebrate coproliths; indeterminate isotropic particles; shell fragments; charcoal (both woody and non-woody tissue); burnt plant pseudomorphs; ash and ash intraclasts; quartz debitage (Supplementary Fig. 2).

### Paleomagnetism

A sub-sample of the continuous column sample from PYS was transported to The Australian Archaeomagnetism Laboratory for preparation and analysis. Once in the laboratory samples were dried to standardize water content, crushed with a non-magnetic mortar, and pestle to become homogenized and then packed into standard 8cc palaeomagnetic plastic cubes. The approach to the analysis follows that of Herries^48^ and a number of analyses were run to establish the magnetic mineralogy of the samples. These included mass specific low-field susceptibility (χlf), mass specific high-field susceptibility (χhf), mass specific frequency dependant susceptibility (χfd) and saturation isothermal remanent magnetization acquisition curves and backfields. This was done to understand the mineralogy driving magnetic susceptibility change through identifying ferrimagnetic vs. anti-ferromagnetic minerals on the basis of their coercivity, establish the concentration of magnetic minerals present within each sample, and examine grain size and domain state trends in the sequence.

The magnetic susceptibility of each sample was measured using a Bartington MS2 susceptibility-meter connected to an MS2B sensor. Samples were measured at 0.47 kHz (low, χlf) and 4.7 kHz (high, χhf) to attain both low and high frequency susceptibility values. These measurements were then used to compute the frequency dependence of the magnetic susceptibility (χfd) and then expressed as a percentage (χfd%) using the formula stated by Dearing et al.^49^. Isothermal remanent magnetizations (IRM) were induced up to 1 T (representing the saturation IRM: SIRM) using a magnetic measurements pulse magnetizer (MMPM10) and measurements made on an AGICO JR6 magnetometer. Forward-field measurements were taken at 20, 500, 600 mT, and 1 T and back-field measurements at 20, 40, 100, 150, 200, 250, and 300 mT. Full IRM acquisition curves and backfields were also produced for each layer of the site. HIRM measurements were also taken using the method described by Liu et al.^50^. Soft IRM measurements showing the concentration of ferrimagnetic minerals were taken at 20 mT. S-ratios (IRM-300 mT/SIRM and IRM-100 mT/SIRM) were calculated to establish variation in the grain size of ferromagnetic minerals. Remanence of coercivity values were also calculated to further establish the mineralogy, grain size, and domain states of the magnetic minerals present within the samples.

### Charcoal abundance

From the continuous column sample described in SM3, subsamples of 1 cm^3^ were extracted for charcoal abundance analysis. The addition of sodium hexametaphosphate to a beaker containing the sample was used to disaggregate the samples and aid in the separation of the organic material and the clay particles^51^. The contents of the beaker was then passed through a 125 μm sieve and the trapped content transferred to a gridded Petri dish. Pieces of charcoal were identified using comparative collections and the total charcoal count was determined through visual inspection and manipulation with a metal probing needle under a Zeiss Stemi 2000-C optical stereomicroscope (×10–40 magnifications). The microscopic charcoal size identified represents charcoal that was locally produced during fires within the catchment area of the site^52^.

### Radiocarbon dating

Items selected for radiocarbon dating were a human bone and a charred sorghum seed (identified by Alison Crowther) from the upper part of the sequence; as well as unidentified charcoal, and ostrich eggshell pieces including a bead, from the middle part of the sequence. These dating samples were either recovered during excavation or taken from the section at the end of excavation.

Fourteen ^14^C measurements were produced at the Oxford Radiocarbon Accelerator Unit (ORAU) (*n* = 10) and by Beta Analytic (*n* = 4). Most charcoal samples were prepared using the standard acid–base–acid (ABA) protocol^53^. While for the younger material (<30 ka BP) this is usually sufficient, it has been shown that ABA does not efficiently decontaminate older charcoal samples when compared with the more rigorous protocol: acid–base oxidation/stepped combustion (ABOx-SC)^54^. Paired ABA and ABOx-SC preparation was used on one sample from context 413 C. For this particular sample, ABA and ABOx methodologies produced identical AMS ages. This is probably due to the exceptional state of preservation of the charcoal in this part of the sequence as well as due to the fact that it is not very old sample. For material from lower levels where only ABA dates from Beta Analytic exist these should be considered minimum ages only. This is demonstrated by the much older ages returned from ostrich eggshell samples from similar contexts.

It is not possible to interpret radiocarbon ages reliably without calibration, due to variation in the concentration of radiocarbon in the atmosphere through time. All terrestrial ^14^C measurements were calibrated using IntCal13 and SHCal13, the most recent internationally agreed calibration curves available^55, 56^. As PYS lies close to the equator, a 68.2/31.8 northern/southern hemisphere split was used in the calibration curve, taking into account the position of the site relative to the inter-tropical convergence zone. The radiocarbon ages obtained from PYS are summarized in Supplementary Table 1.

### OSL

Optically stimulated luminescence (OSL) samples were obtained by hammering metal tubes into section faces following cleaning. The samples were sealed using adhesive tape. Following transport to the Royal Holloway University of London Luminescence Laboratory, samples were processed under subdued orange light. The outer 5 cm of sample (presumed as being exposed to sunlight) was removed and retained for background radioisotope concentration determination.

Quartz was extracted from the part of each sample not exposed to sunlight following burial. As the bedrock at PYS is primarily composed of carbonate, samples were initially wet-sieved to isolate the 212–180 µm size fraction. This removes large bedrock clasts from the sample before acid treatment, meaning that the possibility of incorporating grains liberated by dissolution of the bedrock was minimized.

The volume of 1 M HCl and H_2_O_2_ were used to remove carbonates and organic matter from the 212–180 µm fraction, respectively. The samples were then re-sieved at 180 µm and quartz extracted from the >180 µm fraction using density separations at 2.62 and 2.70 g/cm^3^ followed by a HF acid etch (23 M HF for 60 min followed by 10 M HCl rinse). The resulting, etched samples were sieved at 150 µm to remove partially dissolved grains. All samples were then stored in opaque containers prior to measurement.

All OSL measurements were carried out using a Risø TL/OSL-DA-15 automated dating system^57^, fitted with a single-grain OSL attachment^58, 59^. Single-grains were stimulated using a 10 mW Nd: YVO4 solid-state diode-pumped green laser (532 nm) focused to yield a nominal power density of 50 W/cm^2^ ^57^. All infrared (IR) stimulation was carried out using an IR (870 nm) laser diode array yielding a power density of 132 mW/cm^2^. OSL passed through 7.5 mm of Hoya U-340 filter and was subsequently detected using an Electron Tubes Ltd 9235QB15 photomultiplier tube.

Irradiation was carried out using a 40 mCi 90 Sr/90Y beta source providing ~6 Gy/min. This source is calibrated relative to the National Physical Laboratory, Teddington 60Co γ-source (Hotspot 800)^60^. Due to the spatial inhomogeneity of beta emitters across the active face of our 90 Sr/90Y beta source we calibrated the dose rate to each individual grain position on a single-grain disc^61^ using the method reported by Armitage et al.^62^. For more detail on measurement and quality criteria see Supplementary Note 3.

### Bayesian methods

The absolute age determinations were used to construct an age model using Bayesian software (OxCal 4.3^63^) and the INTCAL13 curve. The determinations were input as values in fraction modern (fM) plus or minor fM errors at 1σ (R_F14C in OxCal). In order to determine whether there are problematic determinations that do not agree with the prior framework, an outlier detection method was applied. When there is a lack of agreement with the prior framework, significant outlier results allow us to quantify the degree of difference. Values excessively higher than the prior outlier probabilities applied are automatically down-weighted in the models. A posterior outlier probability of 0.5 means that the radiocarbon likelihood of the sample is only included in half of the runs of the model. The two Beta dates were assigned a 0.3 value to reflect the inadequate chemical protocol applied in the decontamination of these samples prior to measurement.

Only age estimates older than 20,000 years ago were included in the model. The inclusion of younger ages does not affect the older ages at all. Younger ages were excluded because of the large span of the dates; the model runs with a broad resolution of 50–100 years for the older data, whereas 20 years would be more appropriate for the younger data. In each Bayesian model, a start and end boundary was added in order to bracket the archeological phases within the sequence. The posterior distributions of these boundaries facilitated determination of probability distribution functions (PDF) for the beginning and ending of these phases of activity. Due to the presence of a depositional hiatus represented in the later part of Layer 17, we used two boundaries between the end of 17 and start of Layer 16. In addition, due to uncertainties related to the reliability of sample OSL-7 (see text above) these age estimates were not included in the model.

### Lithics

A total of 30,420 lithic artifacts were recovered through the excavation seasons at PYS between 2010 and 2013. The analyses proceeded by classifying all lithics in accordance with stratigraphic context, raw-material type, and technological class. All artifacts were subsequently weighed and counted according to these categories. Cores and retouched artifacts were further classified in accordance with reduction strategy and typology. For unretouched flakes, blades, Levallois flakes, and bipolar flakes were counted. Levallois^64^, bipolar^65^, laminar, and discoidal strategies as well as resultant blanks were all documented to varying frequencies in a number of the layers.

### Beads, osseous artifacts, and ocher

Over two hundred potential beads, bone tools, engraved bone and stone objects, and pigment lumps recovered during excavation, were examined under a low power reflected light microscope in search for anthropogenic modifications. When necessary, sediment was carefully removed under the microscope with a soft brush or a wet tooth pick. This resulted in the retention of 159 pieces bearing compelling traces of manufacture and use, unmodified or marginally modified shell fragments probably used as beads, and modified and unmodified lumps of iron-rich rock and sediments, possibly used to extract ocher powder.

The retained artifacts were examined at magnifications between ×4 and ×40, and photographed with a motorized Leica Z6 APOA microscope equipped with a Leica Application Suite (LAS) and Multifocus module, and Leica Map DCM 3D computer software. The Multifocus module enables the acquisition of extended depth of field images. Once the digital images had been complete for different heights, algorithms in the software compile them into a single composite images that significant extends the depth of field, and provides clarity in viewing the entire object.

The selected areas of one Conus shell were scanned using a Sensofar Sneox scanning confocal microscope with a ×20 objective. The resulting files were analyzed with Mountains 7.2 software. For further details on the analytical protocols for beads, osseuous artifacts, and ocher see Supplementary Note 4 and the references therein. A full description of all osseous artifacts, beads, and used ocher will be reported elsewhere, but Fig. 3 shows examples of each of these artifact types from PYS excavation, and Supplementary Figure 19 gives an overview of their distribution through the sequence.

### Phytolith analysis

Seventeen sediment samples from the continuous stratigraphic column described in SM3 were processed for phytolith analysis. Of these, the lower six samples were barren. We followed extraction protocols employed on Middle Stone Age sites from adjacent countries^66^ and modern topsoils^67^. The procedure included sieving, drying, deflocculating, acid/base treatment, and sequential density separation by manipulating the specific gravity of sodium polytungstate. Aliquot mounting was with “Entellan New,” which allowed for microscopic inspection (×40) and 3D rotation before drying. The average count per slide was 238 phytoliths. The inferential baseline was grounded on East African phytoliths from plants and soils^68, 69^. Preservation was adequate for morphometric analysis and type identification.

### Mollusk analysis

Macro marine molluskan remains were identified and counted by Patrick Faulkner using comparative modern reference collections. Since no evidence for subsistence on these was found prior to Layer 6, they will be reported in detail elsewhere. PYS is an open-roofed cave with substantial input from the external environment. The paleoenvironmental samples recovered from PYS contained terrestrial mollusk fauna representing the area in and immediately outside the cave over a long time period^70^. There is no evidence that any of these species have been transported to the site through natural or anthropogenic processes.

Excluding large marine shells that form part of the archeological record, the aquatic component of the assemblage is extremely small and is less significant than that seen in faunas from the modern coastal forests on Zanzibar. As a result, transport by fluvial activity or regular flooding of the area can be ruled out. The diverse community of snails could not have been sustained in a closed cave environment but would have required continual external input in the form of leaf litter and the snails themselves or their shells. The non-marine mollusk record from PYS therefore offers a long record of the local environment through time.

### Tetrapod analysis

The zooarchaeological analysis of PYS osseous remains took place in 2012 (Trench 3) and 2014 (Trench 4) and produced a database of 5256 identified specimens (Number of Identified Specimens, NISP). A total of 6.4 kg of bone was excavated from Trench 3 and 14.7 kg from Trench 4. The Trench 4 study included both taxonomic identification and taphonomic analysis, but microfauna were not analyzed in Phase 1 due to time constraints. In Trench 3, microfauna were identified to a very general level (e.g., “Muridae”).

Initial sorting of faunal remains created up to three categories: first, maximally identifiable (maxID) specimens include teeth and those bones that preserve at least one articular surface and/or key landmark, enabling identification to element and usually to taxon or at least taxonomic group (e.g., “bird,” “small carnivore”). MaxID specimens were identified in all contexts in both trenches. Second, minimally identifiable (minID) bones include limb shafts and axial fragments that can generally be identified at least to carcass size; these were identified in nine high-priority contexts from Trench 4, in order to obtain a sample for taphonomic analysis. Third, nonidentified (NID) bones were separated from minID specimens and weighed in these selected contexts. On average across all contexts in Trench 4, 10% of the assemblage was maximally identifiable. When minID specimens identified in nine selected contexts were included the average identification rate rose to 37%.

Taxonomic identifications were made on the basis of extensive reference collections housed at the National Museums of Kenya (Nairobi) Osteology Unit. Calculations of the minimum number of individuals (MNI) were made using the resulting database following completion of this analysis and took into account specimen laterality, size, and where relevant, age estimates. It should be stressed that limb shafts were only studied in selected contexts and therefore could not be used to calculate the minimum number of elements (MNE) or the resulting MNI values for layers or phases, as is standard practice in contexts where density-mediated attrition has occurred. It is therefore possible that MNI estimates will be too low in some instances.

### Stable carbon and oxygen isotope analysis of mammalian tooth enamel

Stable isotope analysis of mammalian tissues has frequently been used to assess the diets and ecologies of East African fossil fauna^71–73^. This work primarily relies on the distinction between the C_3_- or C_4_-photosynthetic pathways at the base of East African foodwebs. In the context of tropical and sub-tropical forest ecologies, this distinction can be used to assess the degree of faunal reliance on C_3_ forest resources as opposed to C_4_ plant resources available in open habitats, with C_4_ plants being enriched in ^13^C relative to C_3_ plants^74–76^.

This distinction is further enhanced by the “canopy effect” whereby vegetation growing under a closed forest canopy is strongly depleted in ^13^C (with correspondingly lower measured δ^13^C) due to low light and the presence of large amounts of respired CO_2_^77, 78^. This results in the tissues of animals consuming forest vegetation, as well as forest herbivores, having lower δ^13^C values than animals pending some, or all, of their time consuming open-habitat foodstuffs e.g.^79^.

Stable oxygen isotope measurements from mammalian enamel can yield further paleoecological information about water and food^80, 81^. Given a constant source of water, plant water δ^18^O will primarily reflect the impacts of relative humidity on leaf water evapotranspiration, with decreasing humidity resulting in increased δ^18^O values^82–84^. In a tropical or sub-tropical setting, increased forest cover, and the resulting shade and increased humidity, will lead to decreased evapotranspiration and therefore decreased δ^18^O, especially on the forest floor^79^. As faunal tooth enamel δ^18^O primarily reflects water and food-water δ^18^O, herbivores feeding and drinking in forests can be expected to have lower enamel δ^18^O than those feeding in open, irradiated areas. This is complicated by physiological and behavioral variables^85^. Nevertheless, animals consuming plants and water in a shaded, forested setting will broadly reflect the corresponding lower levels of evapotranspiration in their enamel δ^18^O.

Stable carbon and oxygen isotope analysis of faunal tooth enamel excavated from the various archeological Phases at PYS was undertaken in order to directly assess the diets and ecologies of animals being exploited by humans living at the site at different points in time. This should, in turn, provide information regarding fluctuations in the degree of forest cover surrounding PYS in the past. Faunal enamel samples were taken from all available PYS Layers. A broad selection of species was sampled for each Layer based on availability. Where possible, up to five members of each species/genus were sampled per layer grouping (Supplementary Data 1). Faunal samples were identified to species and/or genus level using the substantial reference collection available at the National Museums of Kenya, Nairobi. The full list of faunal samples and tooth identifications analyzed in this study are shown in Supplementary Data 1.

Air-abrasion was used to remove any adhering detrital material from the teeth or tooth fragments to be studied. Gentle abrasion with a diamond-tipped drill was performed along the full length of the buccal surface of the tooth or tooth fragment in order to maximize the period of formation represented by the resulting isotopic analysis. The resulting enamel powder was pretreated using standard, published protocols in order to remove any organic or secondary carbonate contaminates. This consisted of a wash in 1.5% sodium hypochlorite for 60 min, followed by three rinses in purified H_2_O. A volume of 0.1 M acetic acid was then added for 10 min prior to another three rinses in purified H_2_O^86, 87^.

Gases were evolved from the treated samples using 100% Phosphoric Acid. t δ^13^C and δ^18^O of the resulting gases was measured using a Thermo Gas Bench 2 connected to a Thermo Delta V Advantage Mass Spectrometer at the Division of Archeological, Geographic and Environmental Sciences Bradford University. δ^13^C and δ^18^O measurements from samples were compared against international standards (NBS 19 and CO-9) registered by the International Atomic Energy Agency (five of each for a run of 60). Replicate analysis of in-house OES and MERCK standards (six of each for a run of 60) suggests that machine measurement error is *c*. ±0.1‰ for δ^13^C and ±0.2‰ for δ^18^O.

Analysis of variance (ANOVA) tests were performed on faunal enamel δ^13^C and δ^18^O to determine whether these isotopic parameters differed between groups of stratigraphic layers. The stratigraphic layers were grouped based on meaningful chronological and stratigraphic divisions as follows: 1–3, 4–6, 7–9, 10–12, 13–16, and 17. Where variance was found to be significant following ANOVA testing, a post-hoc Tukey pair-wise comparison was performed in order to determine which layer groupings were significantly different from each other in terms of δ^13^C and δ^18^O. All statistical analyses were conducted using the free program R software^88^.

### Data availability

The authors declare that all data supporting the findings of this study are available upon request from the authors. The artifacts and faunal remains from the Panga ya Saidi excavation are curated in the National Museum of Kenya, Nairobi, under the site code PYS and the suffixes 10, 11, and 13 (denoting the year of excavation).

## Electronic supplementary material

Supplementary Information

Description of Additional Supplementary Files

Supplementary Data 1

Supplementary Data 2

Supplementary Data 3

## References

[1] Goodwin A, vanRiet Lowe C (1929). The Stone Age cultures of South Africa. Ann. South Afr. Mus..

[2] Lombard M (2011). Quartz-tipped arrows older than 60 ka: further use-trace evidence from Sibudu, KwaZulu-Natal, South Africa. J. Archaeol. Sci..

[3] Henshilwood CS (2012). Late Pleistocene techno-traditions in southernAfrica: a review of the Still Bay and Howiesons Poort, c. 75–59 ka. J. World Prehistory.

[4] Wadley L (2015). Those marvellous millennia: the Middle Stone Age of southern Africa. Azania.: Archaeol. Res. Afr..

[5] Jacobs Z (2008). Ages for the Middle Stone Age of southern Africa: implications for human behavior and dispersal. Science.

[6] Ambrose SH (2002). Small things remembered: origins of early microlithic industries in sub‐Saharan Africa. Archeol. Pap. Am. Anthropol. Assoc..

[7] Klein RG (2000). Archeology and the evolution of human behavior. Evolut. Anthropol. Issues News Rev..

[8] Mellars P (2006). Why did modern human populations disperse from Africa ca. 60,000 years ago? A new model. Proc. Natl Acad. Sci. USA.

[9] Shea JJ, Sisk ML (2010). Complex projectile technology and *Homo sapiens* dispersal into western Eurasia. PaleoAnthropology.

[10] McBrearty S, Brooks AS (2000). The revolution that wasn’t: a new interpretation of the origin of modern human behavior. J. Hum. Evol..

[11] Conard NJ, Will M (2015). Examining the causes and consequences of short-term behavioral change during the Middle Stone Age at Sibudu, South Africa. PLOS One.

[12] Henshilwood CS (2011). A 100,000-year-old ochre-processing workshop at Blombos Cave, South Africa. Science.

[13] Marean CW (2007). Early human use of marine resources and pigment in South Africa during the Middle Pleistocene. Nature.

[14] Marean CW (2010). Pinnacle Point Cave 13B (Western Cape Province, South Africa) in context: the Cape floral kingdom, shellfish, and modern human origins. J. Hum. Evol..

[15] Bouzouggar A (2007). 82,000-year-old shell beads from North Africa and implications for the origins of modern human behavior. Proc. Natl Acad. Sci. USA.

[16] Douka K (2014). The chronostratigraphy of the Haua Fteah cave (Cyrenaica, northeast Libya). J. Hum. Evol..

[17] Gliganic LA, Jacobs Z, Roberts RG, Domínguez-Rodrigo M, Mabulla AZ (2012). New ages for Middle and Later Stone Age deposits at Mumba rockshelter, Tanzania: optically stimulated luminescence dating of quartz and feldspar grains. J. Hum. Evol..

[18] Ambrose SH (1998). Chronology of the Later Stone Age and food production in East Africa. J. Archaeol. Sci..

[19] Brandt SA (2012). Early MIS 3 occupation of Mochena Borago Rockshelter, Southwest Ethiopian Highlands: implications for Late Pleistocene archaeology, paleoenvironments and modern human dispersals. Quat. Int..

[20] Pleurdeau D (2014). Cultural change or continuity in the late MSA/Early LSA of southeastern Ethiopia? The site of Goda Buticha, Dire Dawa area. Quat. Int..

[21] Miller JM, Willoughby PR (2014). Radiometrically dated ostrich eggshell beads from the Middle and Later Stone Age of Magubike Rockshelter, southern Tanzania. J. Hum. Evol..

[22] Diez-Martín F (2009). The MSA/LSA technological transition in East Africa. New data from Mumba Rocksheiter Bed V and their implications in the origin of modern human behaviour. J. Afr. Archaeol..

[23] Groucutt HS (2015). Rethinking the dispersal of *Homo sapiens* out of Africa. Evolut. Anthropol.: Issues, News, Rev..

[24] Faith JT (2015). Paleoenvironmental context of the Middle Stone Age record from Karungu, Lake Victoria Basin, Kenya, and its implications for human and faunal dispersals in East Africa. J. Hum. Evol..

[25] Tryon CA (2016). The Pleistocene prehistory of the Lake Victoria basin. Quat. Int..

[26] Blegen N, Faith JT, Mant-Melville A, Peppe DJ, Tryon CA (2017). The Middle Stone Age after 50,000 years ago: new evidence from the Late Pleistocene sediments of the eastern Lake Victoria basin, Western Kenya. PaleoAnthropology.

[27] Villa P (2012). Border Cave and the beginning of the Later Stone Age in South Africa. Proc. Natl Acad. Sci. USA.

[28] d’Errico F (2012). Early evidence of San material culture represented by organic artifacts from Border Cave, South Africa. Proc. Natl Acad. Sci. USA.

[29] Tryon CA, Faith JT (2013). Variability in the Middle Stone Age of eastern Africa. Curr. Anthropol..

[30] Eren MI, Diez-Martin F, Dominguez-Rodrigo M (2013). An empirical test of the relative frequency of bipolar reduction in Beds VI, V, and III at Mumba Rockshelter, Tanzania: implications for the East African Middle to Late Stone Age transition. J. Archaeol. Sci..

[31] Pargeter J (2016). Lithic miniaturization in Late Pleistocene southern Africa. J. Archaeol. Sci.: Rep..

[32] Tryon CA (2018). Middle and Later Stone Age chronology of Kisese II rockshelter (UNESCO World Heritage Kondoa Rock-Art Sites), Tanzania. PLOS One.

[33] Blome MW, Cohen AS, Tryon CA, Brooks AS, Russell J (2012). The environmental context for the origins of modern human diversity: a synthesis of regional variability in African climate 150,000–30,000 years ago. J. Hum. Evol..

[34] Moernaut J (2010). The seismic-stratigraphic record of lake-level fluctuations in Lake Challa: hydrological stability and change in equatorial East Africa over the last 140kyr. Earth Planet. Sci. Lett..

[35] Tryon CA, Faith JT (2016). A demographic perspective on the Middle to Later Stone Age transition from Nasera rockshelter, Tanzania. Philos. Trans. R. Soc. B.

[36] O’Connor S, Louys J, Kealy S, Samper Carro SC (2017). Hominin dispersal and settlement east of Huxley’s Line: the role of sea level changes, island size, and subsistence behavior. Curr. Anthropol..

[37] Mercader J (2002). Forest people: the role of African rainforests in human evolution and dispersal. Evol. Anth..

[38] Roberts P (2015). Direct evidence for human reliance on rainforest resources in late Pleistocene Sri Lanka. Science.

[39] Fu Q (2014). Genome sequence of a 45,000-year-old modern human from western Siberia. Nature.

[40] Fiedel SJ, Kuzmin YV (2007). Radiocarbon date frequency as an index of intensity of Paleolithic occupation of Siberia: did humans react predictably to climate oscillations?. Radiocarbon.

[41] Lombard M, Parsons I (2011). What happened to the human mind after the Howiesons Poort?. Antiquity.

[42] d’Errico F (2017). Identifying early modern human ecological niche expansions and associated cultural dynamics in the South African Middle Stone Age. Proc. Natl Acad. Sci. USA.

[43] Goldberg, P. & Macphail, R. *Practical and Theoretical Geoarchaeology* (Blackwell Publishing Ltd, Hoboken, NJ, 2006).

[44] Tucker, M. E. *Sedimentary Rocks in the Field: A Practical Guide*(John Wiley & Sons, Chichester, 2011).

[45] Ayala, G., Canti, M., Heathcote, J., Sidell, J. & Usai, M. *Geoarchaeology: Using Earth Sciences to Understand the Archaeological Record* (English Heritage, Swindon, 2007).

[46] Bullock, P., Fedoroff, N., Jongerius, A., Stoops, G.. & Tursina, T. *Handbook for Soil Thin Section Description* (Waine Research, Albrighton, 1985).

[47] Stoops, G. *Guidelines for Analysis and Description of Soil and Regolith Thin Sections* (Soil Science Society of America Inc., Madison, WI, 2003).

[48] Herries AI (2006). Archaeomagnetic evidence for climate change at Sibudu Cave. South. Afr. Humanit..

[49] Dearing JA (1996). Frequency-dependent susceptibility measurements of environmental materials. Geophys. J. Int..

[50] Liu, Q. et al. Environmental magnetism: principles and applications. *Reviews of Geophysics* **50**, RG4002 (2012).

[51] Bamber R (1982). Sodium hexametaphosphate as an aid in benthic sample sorting. Mar. Environ. Res..

[52] Lynch JA, Clark JS, Stocks BJ (2004). Charcoal production, dispersal, and deposition from the Fort Providence experimental fire: interpreting fire regimes from charcoal records in boreal forests. Can. J. For. Res..

[53] Brock F, Higham T, Ditchfield P, Bronk Ramsey C (2010). Current pretreatment methods for AMS radiocarbon dating at the Oxford Radiocarbon Accelerator Unit (ORAU). Radiocarbon.

[54] Bird M (1999). Radiocarbon dating of “old” charcoal using a wet oxidation, stepped-combustion procedure. Radiocarbon.

[55] Reimer PJ (2013). IntCal13 and Marine13 radiocarbon age calibration curves 0–50,000 years cal BP. Radiocarbon.

[56] Hogg AG (2013). SHCal13 Southern Hemisphere calibration, 0–50,000 years cal BP. Radiocarbon.

[57] Bøtter-Jensen L, Andersen C, Duller G, Murray A (2003). Developments in radiation, stimulation and observation facilities in luminescence measurements. Radiat. Meas..

[58] Duller G, Bøtter-Jensen L, Murray A (2003). Combining infrared-and green-laser stimulation sources in single-grain luminescence measurements of feldspar and quartz. Radiat. Meas..

[59] Duller G, Bøtter-Jensen L, Murray A, Truscott A (1999). Single grain laser luminescence (SGLL) measurements using a novel automated reader. Nucl. Instrum. Methods Phys. Res. Sect. B: Beam Interact. Mater. At..

[60] Armitage S, Bailey R (2005). The measured dependence of laboratory beta dose rates on sample grain size. Radiat. Meas..

[61] Ballarini M, Wintle A, Wallinga J (2006). Spatial variation of dose rate from beta sources as measured using single grains. Anc. TL.

[62] Armitage SJ (2011). The southern route “out of Africa”: evidence for an early expansion of modern humans into Arabia. Science.

[63] OxCal version 4.3.2 (2017).

[64] Boëda, E. *Le concept Levallois, variabilité des méthodes* (CNRS éditions, Paris, 1994).

[65] de la Peña P (2015). A qualitative guide to recognize bipolar knapping for flint and quartz. Lithic Technol..

[66] Mercader J, Bennett T, Esselmont C, Simpson S, Walde D (2013). Phytoliths from Middle Stone Age habitats in the Mozambican Rift (105–29 ka). J. Hum. Evol..

[67] Mercader J, Bennett T, Esselmont C, Simpson S, Walde D (2011). Soil phytoliths from miombo woodlands in Mozambique. Quat. Res..

[68] Mercader J, Bennett T, Esselmont C, Simpson S, Walde D (2009). Phytoliths in woody plants from the Miombo woodlands of Mozambique. Ann. Bot. (Lond.)..

[69] Mercader J (2010). Poaceae phytoliths from the Niassa Rift, Mozambique. J. Archaeol. Sci..

[70] Hunt, C. O. & Hill, E. A. in *Molluscs in Archaeology: Methods, Approaches and Applications* Vol. 3 (ed. Michael J. Allen) 100–110 (Oxbow Books, Oxford, 2017).

[71] Levin NE, Simpson SW, Quade J, Cerling TE, Frost SR (2008). Herbivore enamel carbon isotopic composition and the environmental context of *Ardipithecus* at Gona, Ethiopia. Geol. Soc. Am. Spec. Pap..

[72] Cerling TE (2011). Diet of *Paranthropus boisei* in the early Pleistocene of EastAfrica. Proc. Natl Acad. Sci. USA.

[73] Uno KT (2011). Late Miocene to Pliocene carbon isotope record of differential diet change among East African herbivores. Proc. Natl Acad. Sci. USA.

[74] Lee-Thorp JA, Van Der Merwe NJ, Brain C (1989). Isotopic evidence for dietary differences between two extinct baboon species from Swartkrans. J. Hum. Evol..

[75] Kingston JD, Harrison T (2007). Isotopic dietary reconstructions of Pliocene herbivores at Laetoli: Implications for early hominin paleoecology. Palaeogeogr. Palaeoclimatol. Palaeoecol..

[76] Lee-Thorp, J. & Sponheimer, M. in *Handbook of Paleoanthropology* (eds Winifried Henke & Ian Tattersall) 441–464 (Springer, Berlin, 2015).

[77] Farquhar GD, Ehleringer JR, Hubick KT (1989). Carbon isotope discrimination and photosynthesis. Annu. Rev. Plant. Biol..

[78] van der Merwe NJ, Medina E (1991). The canopy effect, carbon isotope ratios and foodwebs in Amazonia. J. Archaeol. Sci..

[79] Cerling TE, Hart JA, Hart TB (2004). Stable isotope ecology in the Ituri Forest. Oecologia.

[80] Koch PL, Michener R, Lajtha K (2007). Isotopic study of the biology of modern and fossil vertebrates. Stable Isot. Ecol. Environ. Sci..

[81] Krigbaum J, Berger MH, Daegling DJ, McGraw WS (2013). Stable isotope canopy effects for sympatric monkeys at Taï Forest, Côte d’Ivoire. Biol. Lett..

[82] Flanagan LB, Comstock JP, Ehleringer JR (1991). Comparison of modeled and observed environmental influences on the stable oxygen and hydrogen isotope composition of leaf water in *Phaseolus vulgaris* L. Plant. Physiol..

[83] Yakir D, Berry J, Giles L, Osmond CB (1994). Isotopic heterogeneity of water in transpiring leaves: identification of the component that controls the δ^18^O of atmospheric O_2_ and CO_2_. Plant Cell Environ..

[84] Sheshshayee M (2005). Oxygen isotope enrichment (Δ^18^O) as a measure of time-averaged transpiration rate. J. Exp. Bot..

[85] Levin NE, Cerling TE, Passey BH, Harris JM, Ehleringer JR (2006). A stable isotope aridity index for terrestrial environments. Proc. Natl Acad. Sci. USA.

[86] Sponheimer M (2005). Hominins, sedges, and termites: new carbon isotope data from the Sterkfontein valley and Kruger National Park. J. Hum. Evol..

[87] Lee-Thorp J (2012). Isotopic evidence for an early shift to C4 resources by Pliocene hominins in Chad. Proc. Natl Acad. Sci. USA.

[88] R Core Team. 2013. R: A language and environment for statistical computing. R Foundation for Statistical Computing, Vienna, Austria.

