# Publisher Correction: 78,000-year-old record of Middle and Later Stone Age innovation in an East African tropical forest

**DOI:** 10.1038/s41467-018-04753-0

**Published:** 2018-06-05

**Authors:** Ceri Shipton, Patrick Roberts, Will Archer, Simon J. Armitage, Caesar Bita, James Blinkhorn, Colin Courtney-Mustaphi, Alison Crowther, Richard Curtis, Francesco d’ Errico, Katerina Douka, Patrick Faulkner, Huw S. Groucutt, Richard Helm, Andy I. R. Herries, Severinus Jembe, Nikos Kourampas, Julia Lee-Thorp, Rob Marchant, Julio Mercader, Africa Pitarch Marti, Mary E. Prendergast, Ben Rowson, Amini Tengeza, Ruth Tibesasa, Tom S. White, Michael D. Petraglia, Nicole Boivin

**Affiliations:** 1 McDonald Institute for Archaeological Research, University of Cambridge, Downing Street Cambridge, CB2 3DZ Cambridge, UK; 2 British Institute in Eastern Africa, Laikipia Road, Kileleshwa, Nairobi Kenya; 3 Centre of Excellence for Australian Biodiversity and Heritage, Australian National University, Canberra, ACT 2601 Australia; 4 Department of Archaeology, Max Planck Institute for the Science of Human History, Kahlaische Strasse 10, Jena, D-07745 Germany; 5 Department of Human Evolution, Max Planck Institute for Evolutionary Anthropology, Deutscher Pl. 6, Leipzig, 04103 Germany; 6 Department of Archaeology, University of Cape Town, Rondebosch, 7701 Western Cape South Africa; 7 Department of Geography, Royal Holloway, University of London, Egham, Surrey TW20 OEX UK; 8 SSF Centre for Early Sapiens Behavior (SapienCe), University of Bergen, Øysteinsgate 3, Postboks 7805, Bergen, 5020 Norway; 9 Malindi Museum, National Museums of Kenya, Malindi, Kenya; 10 Department of Archaeology, Classics and Egyptology, University of Liverpool, 12–14 Abercromby Square, Liverpool, L69 7WZ UK; 11 Department Environment, York Institute for Tropical Ecosystems, University of York, Heslington, York, YO10 5NG UK; 12 School of Social Sciences, The University of Queensland, St Lucia, QLD 4072 Australia; 13 Department of Archaeology and History, The Australian Archaeomagnetism Laboratory, Palaeoscience Labs, La Trobe University, Melbourne Campus, Bundoora, VIC 3086 Australia; 14 UMR 5199 PACEA, CNRS/Université de Bordeaux, Bâtiment B18, Allée Geoffroy Saint Hilaire, CS, 50023 - 33615 PESSAC CEDEX, France; 15 Research Laboratory for Archaeology and the History of Art, Dyson Perrins Building, South Parks Road, Oxford, OX1 3QY UK; 16 Faculty of Arts and Social Sciences, Department of Archaeology, The University of Sydney, Sydney, NSW Australia; 17 School of Archaeology, University of Oxford, 36 Beaumont Street, Oxford, OX1 2PG UK; 18 Canterbury Archaeological Trust, 92A Broad Street, Canterbury, Kent CT1 2LU UK; 19 Coastal Forests Conservation Unit, National Museums of Kenya, Kilifi, Kenya; 20 Centre for Open Learning, University of Edinburgh, Paterson’s Land, Edinburgh, EH8 8AQ Scotland UK; 21 Biological and Environmental Sciences, University of Stirling, Stirling, FK9 4LA Scotland UK; 22 Department of Anthropology and Archaeology, University of Calgary, 2500 University Drive, Calgary, AB T2N 1N4 Canada; 23 Grup de Recerca Aplicada al Patrimoni Cultural (GRAPAC), Department of Animal Biology, Plant Biology and Ecology, Faculty of Biosciences, Autonomous University of Barcelona (UAB), Campus Bellaterra, Bellaterra, 08193 Spain; 24 Department of Sociology and Anthropology, Saint Louis University, Avenida del Valle 34, Madrid, 28003 Spain; 25 Invertebrate Biodiversity, National Museum Wales, Cathays Park, Cardiff, CF10 3NP UK; 26 Department of Anthropology and Archaeology, University of Pretoria, cnr Lynnwood Road and Roper Street, Hatfield, South Africa; 27 University Museum of Zoology, Cambridge Downing Street, Cambridge, CB2 3EJ UK; 28 Department of Life Sciences, The Natural History Museum, Cromwell Road, London, SW7 5BD UK; 29 Human Origins Program, Smithsonian Institution, Washington, DC 20560 USA

Correction to: *Nature Communications* 10.1038/s41467-018-04057-3, published online 09 May 2018.

The originally published version of this article contained an error in Fig. 3, whereby an additional unrelated graph was overlaid on top of the magnetic susceptibility plot. Furthermore, the Article title contained an error in the capitalisation of ‘Stone Age’. Both of these errors have now been corrected in both the PDF and HTML versions of the Article.

