# Supplementary material for: 78,000-year-old record of Middle and Later Stone Age innovation in an East African tropical forest
Source: Nat Commun. 2018 May 9;9:1832. doi: 10.1038/s41467-018-04057-3 (PMC5943315; doi:10.1038/s41467-018-04057-3)
Supplement: Supplementary file 1 — Supplementary Information [file 41467_2018_4057_MOESM1_ESM.pdf]

Supplementary Information

78,000-year-old record of Middle and Later Stone Age innovation in an East African tropical forest

Supplementary Note 1: The local environment

<u>Geological and geomorphic setting</u>

Panga ya Saidi, in the Nyali Coast of southern Kenya, is situated about 15km from the shore, at an altitude of about 150m. It is a large, multi-storey, partly unroofed cave complex – part of a constellation of caves, rockshelters and collapse dolines that dot the eastern flanks of the Dzitsoni Uplands (Fig. 1). The seafloor drops below a depth of -125m in comparison to modern sea level within 5km of the current shoreline, ensuring the site remain in close proximity to the coast throughout the documented occupation phases (Fig. 1a, Supplementary Fig. 1).

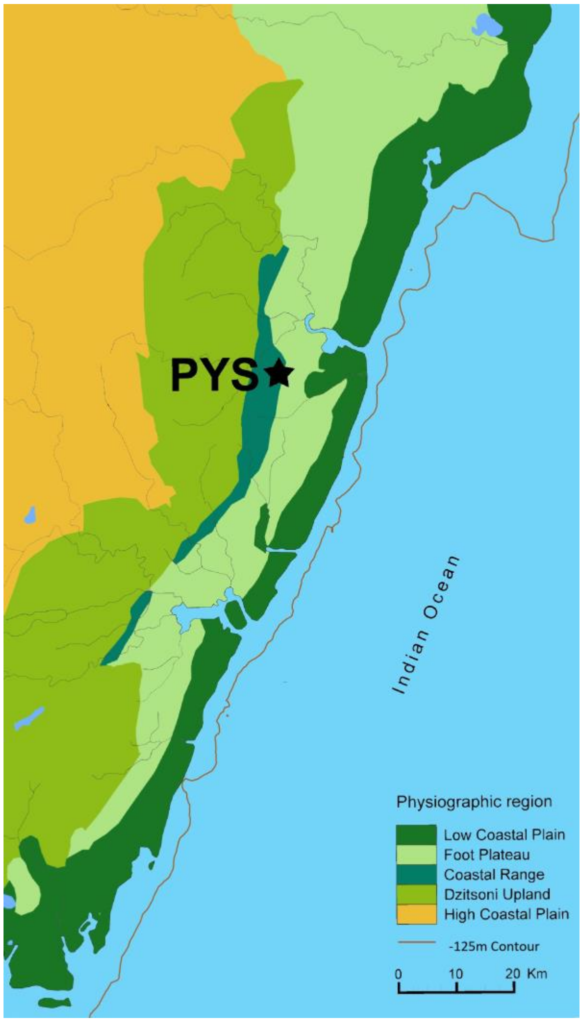

Supplementary Figure 1. Map of the physiographic region in which PYS is located indicating the close proximity of the -125m contour to the modern shoreline along the Nyali Coast.

The Dzitsoni Uplands is a NNE-SSW trending, fault-bounded ridge that forms part of the physiographic transition between the late Quaternary coastal plain, with Late Pleistocene marine terraces transversed by meandering tidal creeks and estuaries, and the elevated flatlands of the extensive, semiarid Nyika Plateau further inland (Fig. 1a, Supplementary Fig. 1). The eastern flanks of the Dzitsoni Uplands, from ca. +240 to +60m, comprise a staircase of near-horizontal surfaces bounded by discontinuous low cliffs and incised by valleys. This part of the coastal lowlands, interpreted as a succession of Tertiary(?)-Quaternary surfaces of subaerial erosion and, in its distal, shoreward part, marine terraces, was termed the *Foot Plateau* by earlier researchers[1,2].

This stepped relief is formed on faulted and gently tilted sedimentary rocks of Mesozoic (Triassic to Jurassic) age, and on terrestrial, marginal marine and shallow marine sediments and soils of Late Tertiary to Holocene age (Supplementary Fig. 2). Carbonate sediments occur as two distinct, coast-parallel stripes: the Mid-Jurassic Kambe Limestone of the Dzitsoni Uplands, wherein Panga ya Saidi and numerous other caves and rockshelters are situated, and various reef and shallow-marine limestones associated with the Pleistocene limestone terraces near the coast. Karstic weathering has been particularly pronounced in these parts of the landscape, with a plethora of surface and subterranean landforms, from pinnacle karst and dolines to extensive, and largely unexplored, multi-storey caves and shallow rockshelters – many of which preserve evidence for Pleistocene habitation.

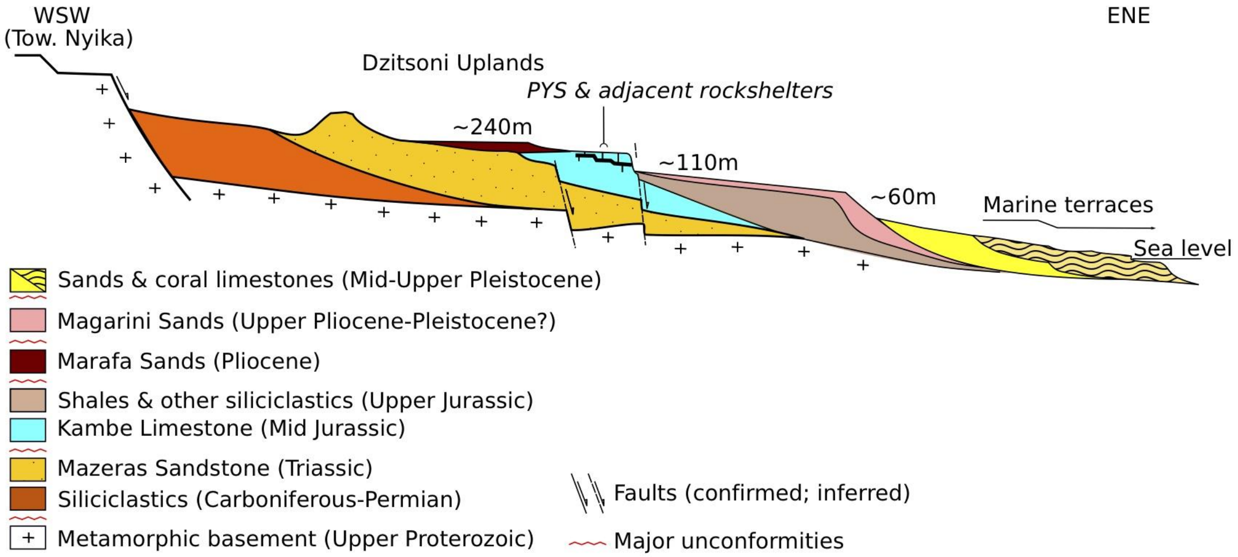

Supplementary Figure 2. Summary geology of Dzitsoni Uplands and the Nyali Coast (not in scale; synthesised from refs[1-5] and Sealinks fieldwork).

Panga ya Saidi is one of the largest known caves in the Dzitsoni Uplands. Although much of the cave if unroofed, a host of speleothems and solutional mesoforms evidence a long and complex evolution, which probably included an early phase of hypogenic speleogenesis. Links between cave formation and evolution on the Dzitsoni caves and the Cainozoic evolution of the East African continental margin are under investigation.

Vegetation and climate

The Nyika Plateau is characterized by a dry Acacia Thorn-Bushland (*Acacia-Euphorbia*). However, moving eastwards to the Dzitsoni Uplands, a greater diversity of plant communities is present. The Dzitsoni vegetation is broadly categorized within the Zanzibar-Inhambane Regional Mosaic, represented by lowland moist and dry forest remnants[6,7]. Plant communities include the Lowland Dry Forest (*Manilkara-Diospyros*) and related Sokoke Forest (*Cynometra–Manilkara*); the Shale Savanna (*Manilkara–Dalbergia*); Lowland 'Miombo' Woodland (*Brachystegia-Afzelia*); Lowland Rain Forest (*Sterculia-Chlorophora/Memecylon*); and Lowland Moist Savanna (*Albizia-Anona/Panicum*). PYS is situated on the fringes of Sokoke Forest (which also extends into the unroofed part of the cave). It overlooks Shale Savanna right in front of the cave and, further east, Lowland Dry Forest on Coral Rag (*Combretum schumannii-Cassipourea*) and Mangrove Thicket (*Ipomoea pes-capre–Rhizophora mucronata*) on the Low Coastal Plain.

The average annual rainfall varies between more than 1400 mm on the Low Coastal Plain, to less than 700 mm on the Nyika Plateau[8]. This is distributed within two rainy seasons: the short rains between October and December, and the long rains between April and June. Modelling the rainfall regimes of the Last Glacial Maximum and the Last Interglacial indicates that the Nyali Coast has consistently received rainfall while the interior has been markedly drier (Supplementary Fig. 3).

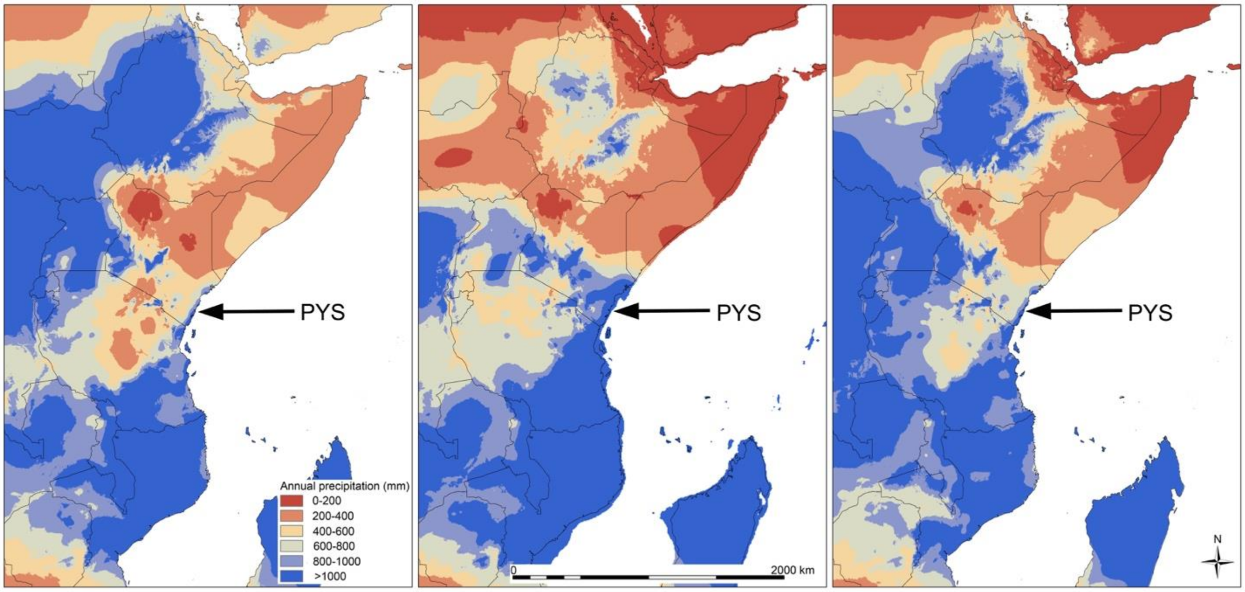

Supplementary Figure 3. Modelled mean annual rainfall in Eastern Africa for the Last Interglacial (left)[9], the Last Glacial Maximum (center)[10], and the present (right)[11].

Supplementary Note 2 Further details of the excavation method, lithostratigraphy, thin section micromorphology, palaeomagnetism and inferred depositional processes

On completion of the 2013 excavation, PYS Trench 4 reached a depth of about 3 m through exclusively detrital deposits (various loams with occasional coarser clasts and, in the upper two thirds of the profile, ashes, ash-derived carbonates, and copious quantities of human occupation debris); resolved into 19 layers (Supplementary Fig. 4). Bioturbation has occurred at various scales, from large (10’s of cm) animal burrows in some layers to tree root channels and microscopic endofaunal galleries, but most stratigraphic boundaries were traceable along the entire profile. Three lithological boundaries divide the profile into four main lithostratigraphic units described below in turn.

<u>Unit I (Layers 19 to 17): Reddish brown clayey (to sandy-clayey) loams with occasional lithics and bone</u>

Reddish brown, cohesive clay loam in the basal >110 cm, coarsening to sandy clayey loam up-profile. Coarser particles include heavily karstified/corroded clasts of bedrock limestone (maximum clast: ca. 10 cm), angular fragments of idiomorphic calcite spar (evidently derived from vugh and joint-filling calcite in bedrock limestone) and rare well preserved, mainly unabraded lithics, mollusk shell and mammalian bone fragments (many degraded and decalcified), concentrated in cm-scale accumulations.

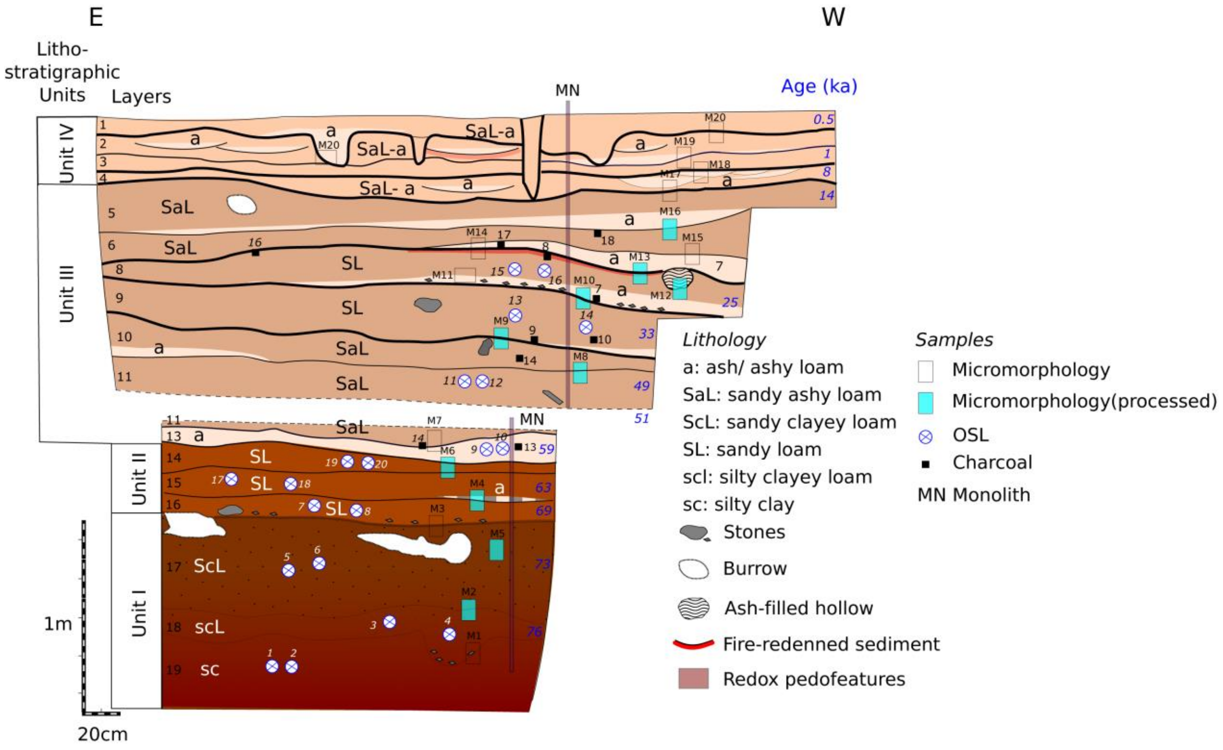

Supplementary Figure 4. Lithostratigraphy and sampling of Trench 4, Panga ya Saidi.

Sesquioxide impregnation of the matrix is pervasive, with numerous redox pedofeatures: orthic and dysorthic/anorthic nodules at various scales (from sand sized spherical pisoliths to mammillate and irregular, evidently *in situ* aggregates. Irregular and mammillate nodules (μ to cm-sized) of microcrystalline calcite are also present; locally these engulf sesquioxide nodules.

Diffuse boundaries and compositional variation (mainly in clay content) may demarcate three intra-unit layers (Supplementary Fig. 4). With the exception of lenses of lithics and inbricated/subhorizontal bone fragments in the basal two layers, Unit I deposits appear structureless internally.

Two petrographic thin sections across Unit I deposits show relatively abundant sand-sized quartz grains (many etched) and rare pedoclasts reworked from outside the cave, pervasive impregnation of the groundmass by sesquioxides, the presence of silt-sized organic particles of unknown thermal history and a scarcity of coarser charcoal or other burning by-products.

Unit I is interpreted as an oxisol developed in colluvial deposits. Fines may have originated in a deep and well developed weathering mantle on the overlying limestone bedrock, consistent with the presence of forest biome above and around the cave. Further eluviation of fines down-profile may have taken place post-depositionally. Redox pedofeatures, indicative of seasonal water saturation, may reflect humid climatic conditions of the later part of MIS 5. Larger clasts evidently resulted from wall disintegration. This unit's advanced pedogenesis is in keeping with the early age inferred form OSL estimates. This unit may be a catenary equivalent of the deep red oxisols present on the overcave surface. Human habitation appears to have been very sporadic in this period.

Unit I/II (Layer 17/16) interface

A moderately sharp compositional boundary separates the clayey oxisol of Unit I from the overlying sandy loams with material culture and human occupation debris of Unit II. This interface is associated with accumulations of small, angular limestone clasts (≤20 cm roof/wall spall) across part of the profile, which may indicate the development of a long-standing cave floor, and a distinct horizon of 10s-of-cm-sized horizontal animal burrows, perhaps reflecting compositional and pore moisture differences across the boundary. It also coincides with a marked departure in magnetic susceptibility parameters, interpreted as a depositional hiatus during the MIS 5 to MIS 4 transition (SM6).

Unit II (Layers 16 to 14): Orange brown silty loam with bedrock clasts and reworked ash

Light brown to orange pebbly silty loam with (locally abundant) limestone clasts and occasional iron/manganese pisoliths in a matrix-supported fabric. Lighter colour, notably lower clay content and (indistinct) subhorizontal bedding, traceable from platy limestone clasts, differentiate this from underlying Unit I. This unit comprises three subhorizontal layers (Supplementary Fig. 4). Human inputs include lithics, bone fragments and charcoal flakes; by the base of Layer 15 these are more abundant than any human inputs in Unit I (Supplementary Fig. 5).

Two petrographic thin sections from this unit show variable quantities of microscopic habitation debris (charcoal, calcined and charred bone splinters, intraclasts of ash, silt-sized microcharcoal)

in a granular/crump groundmass. Granular-crump microstructure is notably more developed in these (and overlying) deposits, suggesting a shift in endofaunal activity above the Unit I/II boundary. Microlayers with aggregates of calcite, charcoal silt, and microscopic bone splinters may have resulted from intensive bioturbation of ash deposits. Pedoclasts of inferred extra-cave origin and a high content in detrital quartz grains (up to 15-20% of thin section surface locally) suggest that much of the mineral content of PYS deposits was washed in from outside.

Unit II is interpreted as a floor colluvium, resulting from the redistribution of wall disintegration clasts, in-washed fines, and coarser grains brought in from the over-cave surface (e.g. iron/manganese pisoliths) by seasonal floor wash and other floor processes. Accumulated roof fall clasts at its base may mark a depositional hiatus. Human habitation contributed moderate amounts of ash and other burning byproducts, while intense bioturbation by endofauna modified sediment texture at the microscopic level.

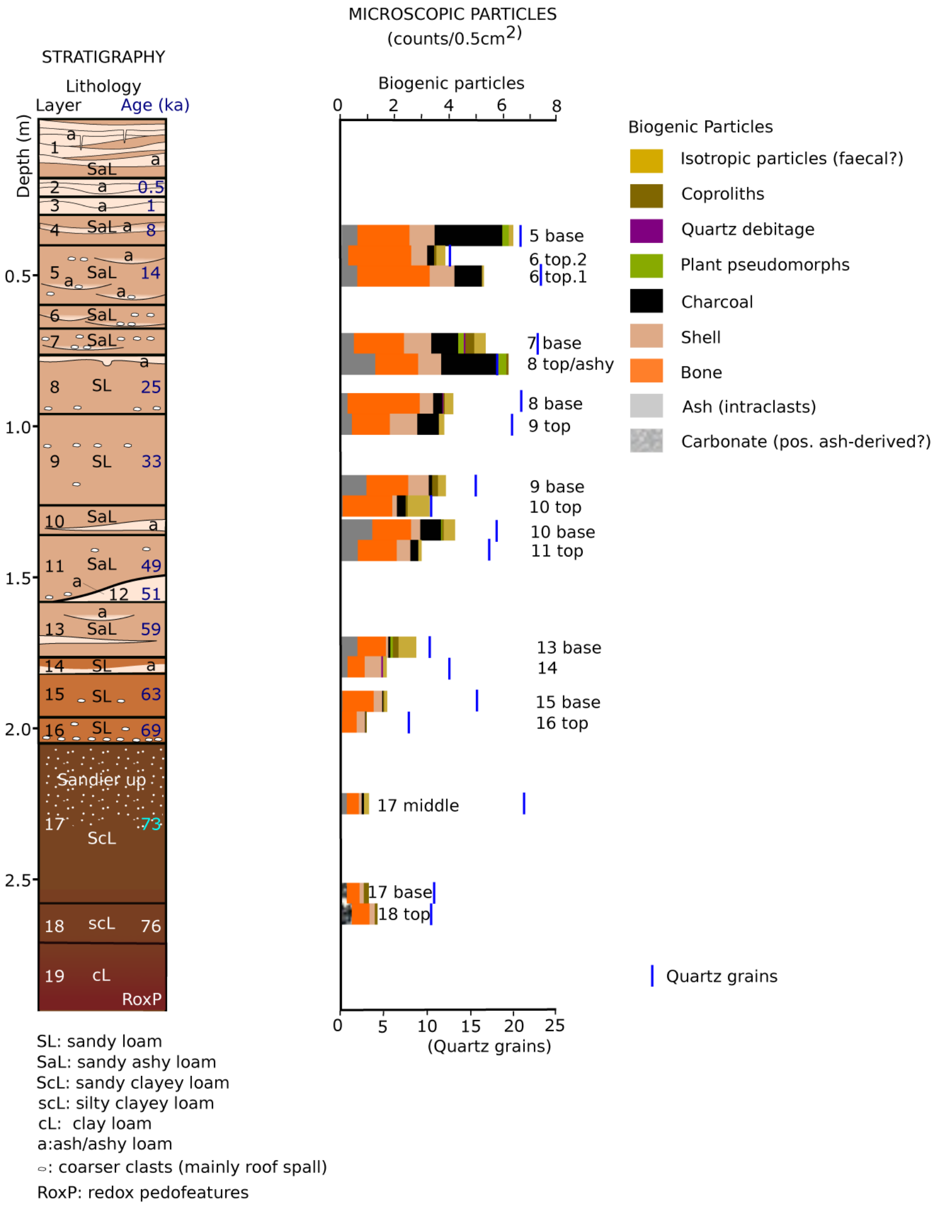

Supplementary Figure 5. Stratigraphy and microscopic particle count in PYS Trench 4.

Unit II/III (Layer 14/13) interface

A sharp surface with some cm of erosional relief separates Unit II from the intercalations of sandy loams and ash layers/lenses with abundant material culture and other occupation debris that constitute the upper ca. 2/3rds of the profile (Unit III).

Unit III (Layers 13 to 5): Beige heterogeneous loams with ash and abundant human inputs

The earlier deposit of this unit is a discontinuous but laterally traceable ash layer (Layer 13) that drapes the Unit II/III interface. Unit III is a heterogeneous, ca. 1.6-1.7 m thick accumulation of soft, light orange to beige sandy silty loams with cm to 10's of cm thick ash layers/lenses, occasional angular limestone clasts (matrix-supported fabric) and a content of lithics, mammalian bone, shell, and charcoal which, albeit variable, is notably higher than in underlying lithostratigraphic units (Supplementary Fig. 5). Depositional architecture consists of ca. 7-30 cm sized beds and lenses bounded by relatively sharp undulating surfaces with shallow cut-and-fill features locally (Supplementary Fig. 1). Many layer boundaries in the lower half of this unit deep gently (15-25°) away from the nearest dripline towards the cave wall (south). Ash layers/lenses vary in thickness, content, internal structure (e.g. finely laminated vs. unstructured) and relationship with surrounding sediments (erosive boundaries; ash as filling of floor microrelief, presence or absence of reddened substratum), permitting the distinction of *in situ* hearths and deposits reworked on the cave floor (e.g. by floor wash; or, possibly, raking).

Several sharp stratigraphic interfaces (palaeofloors with some degree of erosion of underlying deposits; possible depositional hiatuses) are distinguishable within the (largely) human-mediated deposits of Unit III (Supplementary Fig. 4). The Layer 13/12 boundary, with erosional microrelief, may mark a significant hiatus between two occupation phases (as demonstrated by the distribution of OSL date estimates: Supplementary Note 3). A sharp subhorizontal boundary draped with ash deposits separates Layer 10 from 9. The Layer 9 to 8 interface marks a compositional shift towards sediments of higher clastic content: Layer 8, immediately above is notably richer in angular limestone fragments, which impart a clast-supported fabric. This boundary is correlative with a shift in magnetic susceptibility parameters, interpreted as a hiatus (Supplementary Note 6). The sharp boundary between Layers 8 and 6/7– evidently a palaeofloor – is associated with thick ashy deposits above fire-reddened substratum and ash-filled floor hollows (Supplementary Fig. 4).

Seven petrographic thin sections demonstrate the compositional and textural variability and the high concentration of human and other biogenic inputs throughout Unit III deposits. A sharp rise in putative microscopic human inputs (ash, ash intraclasts, microcharcoal, bone splinters) at the very base of this unit indicates intensification of human habitation at this part of the cave. As in underlying deposits, the reworked pedoclasts, quartz sand, ferricrete nodules/angular fragments, and (rare) bedrock limestone and speleothem fragments record the variable relative contribution of colluvial in-wash from outwith the cave and cave wall disintegration in the mineral fraction of PYS.III deposits. Biogenic inputs include bone and excrements from cave fauna (birds, bats, and other small vertebrates) and various human habitation debris (burning by-products, bone, shell, debitage) (Supplementary Fig. 5). A peak in quartz concentration around the Layer 8/7 boundary may record higher colluvial and, possibly, aeolian inputs during the LGM. Inferred depositional

settings include (largely) *in situ* hearths, middens, ash-filled cavities, admixtures of colluvia and burning by-products (from raking and/or inadvertent reworking of hearth deposits on the cave floor) and diverse cave floor accumulations with variable abundance of small vertebrate remains (bones, faecal pellets). Postdepositional change included bioturbation, which imparted a crumb/granular microstructure throughout, and localised dissolution of ash, shell, and bone, as well as deposition of neomorphic phosphates.

Overall, Unit III registers diachronous deposition of in-wash colluvia, roof fall rubble, and various and abundant residues from human occupation. The onset of this unit marks intensification of human occupation at this part of the cave. Higher-order stratigraphic subdivision within the largely human-mediated Unit III deposits appears to reflect predominantly the pulsed, intermittent history of human occupation in this part of the cave.

Unit III/IV (Layer 5/4) interface

Unit PYS.III terminates against a sharp surface with ca. 20 cm of erosive relief about 35-45 cm below datum (Supplementary Fig. 4). A shift in magnetic susceptibility parameters across this boundary is interpreted as a hiatus (see below).

Unit IV (Layers 4 to 1): Beige heterogeneous silty loams with ash and abundant human inputs

The Unit III/IV boundary is overlain with accumulations of angular limestone clasts (up to ca. 7cm; locally imbricated) and lithics – an inferred palaeofloor. Above comes a succession of ca. 7 to 15 cm thick layers of loose, soft, pale beige, silty pebbly ashy loam intercalated with ash layers and lenses. Four continuous layers and many lenses are demarcated by sharp bounding surfaces with 20-30 cm of relief truncating underlying deposits. Charcoal, bone, marine shells and lithics are abundant; Tana Tradition ceramics are also present from Layer 3 upwards.

The uppermost 5-20 cm of the section contain deep, narrow, trough-shaped and cylindrical hollows that extend deep into underlying sediments that are likely animal burrows (Supplementary Fig. 4). These cavities are filled with laminated calcareous ash or admixtures of pale ash and darker, more detrital sediment. *In situ* hearths, with ash (up to ca. 4 cm) and cm sized charcoal fragments above rubified silty substratum (up ca. 2-3 cm of rubification) are also present. Ash layers can be traced laterally to increasingly more diffuse accumulations of pale ash and ash-rich clastic loam, extending for ca. 1-1.5 m.

Unit IV is also interpreted as admixture of human occupation debris – predominantly burning by-products, with colluvial deposits. As in underlying units, sharp interlayer boundaries suggest that deposition was intermittent. The fine, silt to clay-sized calcareous content of Unit PYS.IV loams may have resulted from the reworking of hearth ash by trampling or other human disturbance of the cave floor (e.g. in the course of burial practices), surface wash and rill flow (as evidenced by cm-wide trough shaped deposits), and/or weathering of the cave walls by blue green algae[12,13]. Mechanical degradation of the cave walls contributed coarser clastic input. As in earlier deposits, wall degradation may have been promoted by frequent human presence in the cave.

Paleomagnetism

χLF undergoes a number of very large changes throughout the 276cm sequence (Supplementary Fig. 6), generally decreasing between 280 and 207cm (layers 18-16), and generally increasing above 207cm through layers 16 to 1. The decrease in χLF is much more rapid below 207cm, suggesting a depositional rate change with a more gradual accumulation above 207cm (from Layer 16). Given the known age of the site covering the last 76 ka or so of the last glacial cycle, there are a number of quite obvious features that seem to be driven by climatic variation. Most notably, the high χLF values in layer 18 and the base of 17 are suggestive of the MIS5 interglacial (dated to pre ~71 ka). A major decrease during the upper part of Layer 17 would correlate with the transition to MIS4 dated here to ~70 ka. There is a large jump in the values between Layers 17 and 16, with much lower values in Layer 16. This is suggestive of a major depositional hiatus that likely represents the coldest phase of MIS4. Below this MSA tools are found in the sequence and above LSA occurs, also suggesting major changes in the sequence. The sharp boundary with the underlying sediments, and changes in microstructure and sediment composition across this boundary (identified through micromorphology) are also consistent with a hiatus. Layer 16 above this hiatus is also lighter in colour and notable lower in clay content, indicating another reason for the magnetic mineralogical changes seen. Micromorphology also suggests that deposits in Layers 16 and 15 are an admixture of colluvia with human habitation deposits. This is consistent with the magnetic changes seen and their interpretation as the first pulse of human occupation after the hiatus.

In Layer 16 through 15 χLF increases rapidly as might be expected at the end of MIS4, which would generally be consistent with the age of ~61.5 ka for layer 15. Layer 14 is represented by a major spike in χLF that is likely anthropogenic in nature. Layers 13 to 9 have roughly similar fluctuating values that would be consistent with deposition in MIS3 and these layers are dated to between 61.5 and 33 ka, which would again be consistent with this. Layers 8-5 have high χLF values that again are roughly similar, but fluctuating. These layers are dated to MIS2, between ~25 and 14.5 ka and as such they should have been deposited across the coldest part of the last glacial period, but the χLF values do not entirely seem to reflect this. There is certainly no evidence for a major cold/arid(?) phase that represents the Last Glacial Maximum (LGM). In Layers 4-1 the χLF values are again much higher; with dates of <7 ka, this is consistent with the warmer climates of the Holocene. The rapid increase in values again suggests a hiatus between Layers 5 and 4, sometime around the Pleistocene-Holocene transition. While the χLF sequence is broadly interpretable as a climatically driven sequence that is comparable to marine isotope stages there are some notable discrepancies, like the MIS2 layers. Moreover, the fact that fire use converts and alters iron phases means that in archaeological sites such purely climatically driven interpretations are not viable[14,15]. This is highlighted in particular by Layer 14.

The overall trend of χLF and χFD correlate well and are identical below 207cm: initial high values are followed by a sharp decrease. A sharp drop between 210 and 207cm suggests a major depositional break, which is correlated to the Layer 16-17 contact. Above 207cm χLF increases at a much greater rate than χFD. This suggests that there is also a major change in the magnetic mineralogy at this 207cm hiatus. In contrast χFD% values have an inverse relationship to χLF and χFD, with an increase in χFD% below 200cm, followed by a sharp decrease between ~200 and ~180cm, followed by a much more gradual decrease up through the section.

χFD is a proxy for the presence of ultra-fine grained (<0.01 μm), superparamagnetic (SP) grains that are driven primarily by pedogenic enhancement in the external cave environment due to rainfall changes[16]. It is defined as $\chi FD = \chi LF - \chi HF$[17]. In contrast χFD% (defined as $(\chi LF - \chi HF) / \chi LF \times 100\%$) is controlled by both the grain size distribution of SP to SD particles[18-20],

and by the relative concentration of SP to SD and PSD (pseudo-single domain) particles[16,21]. Herries[14,15] has shown that χFD% is a good marker for anthropogenic alteration with high χLF and high to medium χFD% values representing anthropogenically altered deposits. In-situ fireplaces have very high χLF and overall lower χFD% values.

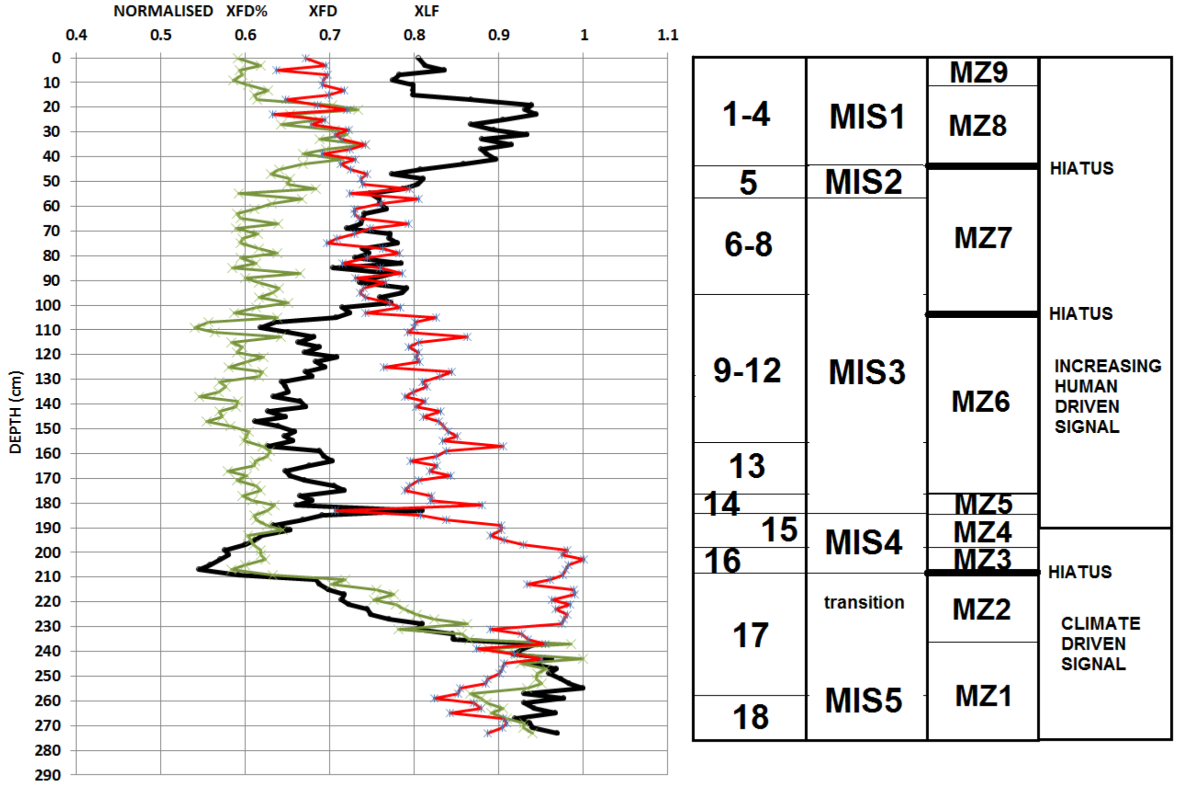

Supplementary Figure 6. Normalized Magnetic Susceptibility parameters (χLF [bulk ferrimagnetic iron oxide content], χFD [environmental signal, SP grains] and χFD% [anthropogenic fire driven signal]) for the PYS sequence compared against stratigraphic layers, marine isotope stages [MIS], magnetic zones (MZ1-9) and inferred hiatuses.

Supplementary Note 3: Full details of dating results and quality control practices

Radiocarbon

Supplementary Table 1. Summary of radiocarbon results from PYS in stratigraphic order. The Beta Analytic samples from Layers 11 and 12 were prepared using ABA not ABOx-SC, so those that returned a finite age (marked with *) may be regarded as minimum ages.

Sample ID	^{14}C pre-treatment	Lab code	^{14}C age	±	Layer	Calendar date	Excavation Context	Material
PYS13-04 403/45	Ultrafiltration	OxA-30803	388	27	Feature between 1 and 2	1450-1650 AD	403	Human bone
PYS10-01, 103D/44	Acid only (fragile)	OxA-29285	1212	23	Layer 3	772-869 AD	103D	Charred seed (*Sorghum bicolor*)
PYS13-5	ABA	OxA-30440	6797	36	Layer 4	7670-7610 BP	Section	Charcoal unidentified
PYS13-6	ABA	OxA-30441	12375	50	Layer 5	14570-14200 BP	Section	Charcoal unidentified
PYS 13-1	Carbonate	OxA-29983	20835	75	Layer 8	25300-25030 BP	412	Ostrich eggshell bead
PYS 13-2a	ABOx-SC	OxA-30146	28970	190	Layer 9	33460-32930 BP	413C	Charcoal unidentified
PYS 13-2b	ABA	OxA-29431	28780	180	Layer 9	33270-32670 BP	413C	Charcoal unidentified
PYS 13-3	Carbonate	OxA-29984	44500	450	Layer 11	48380-47160 BP	415A	Ostrich eggshell
PYS13-04 415A *	ABA	Beta-368059	*39660*	*530*	Layer 11	*43830-42900 BP*	415A	Charcoal unidentified
PYS 13-4a	Carbonate	OxA-29985	45700	500	Layer 11	49770-48660 BP	Section	Ostrich eggshell
PYS 13-4b (duplicate)	Carbonate	OxA-29986	45550	550	Layer 11	49770-48660 BP	Section	Ostrich eggshell
PYS13-04, 415C	Carbonate	Beta-368060	> 43500	–	Layer 11	*> 43500*	415C	Ostrich eggshell
PYS13-04, 416 *	ABA	Beta-368062	*38490*	*460*	Layer 12	*42850-42220 BP*	416	Charcoal unidentified
PYS13-04, 416B	Carbonate	Beta-368063	> 43500	–	Layer 12	*> 43500*	416B	Ostrich eggshell

Optically Stimulated Luminescence

Equivalent doses (De) were determined using the single-aliquot regenerative-dose (SAR) method [22], which allows an independent estimate of De to be generated for each mineral grain measured. The SAR technique involves making a series of paired measurements of OSL intensity. The first measurement in each pair gives the natural (Ln) or regenerated (Lx) OSL intensity, while the

second measurement (Tx) gives the OSL intensity in response to a fixed test dose. Tx is used to monitor changes in sensitivity (luminescence per unit dose) during the measurement sequence. By dividing the natural or regenerated luminescence intensities by their respective test dose intensity (Ln/Tn or Lx/Tx), a sensitivity-corrected luminescence response is determined. While the SAR sensitivity correction often performs well across a wide range of preheating conditions[23,24], it is frequently observed that optimum conditions vary between samples[22,25]. Consequently, the performance of the SAR method was tested prior to adopting measurement conditions for the dating study using dose recovery tests[26,27].

Dose recovery experiments involve controlled bleaching of the natural OSL signal from a group of aliquots, followed by the application of a known laboratory radiation dose (the "known dose"). The equivalent dose for these aliquots is then determined using the SAR method (the "recovered dose"). Where the known and recovered doses are indistinguishable, there are grounds for assuming that the measurement conditions used are appropriate for that sample. A single aliquot dose recovery test was performed on one PYS sample, and indicated that a wide range of commonly adopted preheating regimes yielded acceptable results. We adopted a preheating regime of 220 °C held for 10 s prior to the natural/regenerated dose OSL, and a 160 °C cut heat prior to measurement of the test dose OSL. The suitability of this preheating regime was tested by performing a single-grain dose recovery experiment on grains (n=500-800) from six of the seven samples measured (Supplementary Table 2). No dose recovery experiment was performed on sample OSL7 (Layer 16) since insufficient material was available.

Supplementary Table 2. Dose recovery results for six samples from PYS13 using the preheating regime adopted for single-grain measurements.

Layer	Sample (PYS13-…)	Number of grains (Analysed/accepted)	Known dose (Gy)	Recovered dose (CAM, Gy)	Known/Recovered (ratio)
9	OSL13	800/98	56.7	57.1±0.6	1.01±0.01
11	OSL11	700/72	65.3	63.8±0.7	0.98±0.01
13	OSL9	500/38	79.9	78.4±1.5	0.98±0.02
15	OSL17	800/70	99.5	97.3±1.5	0.98±0.02
17	OSL5	800/52	122	119±2	0.98±0.01
18	OSL3	800/56	108	107±2	1.00±0.02

All ages were produced using single-grain datasets. Measurement conditions, with the exception of regeneration doses, were held constant for all samples. Optical stimulation was carried out at 125 °C for 2 s using the green laser. The OSL signal was that recorded during the first 0.3 s of stimulation, with a background signal from the last 0.3 s of stimulation subtracted[28,29]. Dose response curves were constructed by plotting sensitivity-corrected luminescence response (Lx/Tx) against regeneration dose. Regeneration doses were chosen to bracket the full range of expected equivalent dose values for each sample. A number of additional regeneration points were also included to monitor the quality of the data generated (see below). These were: (1) a zero dose point, to measure "recuperation"; (2) a repeat measurement of the initial regeneration dose, to calculate the "recycling ratio", which tests the internal consistency of the growth curve; (3) a second repeat of the initial regeneration dose followed by a room temperature IR bleach and subsequent OSL measurement, to calculate the "IR depletion ratio", which allows contaminating feldspar grains to be detected. Dose response curves were fitted with a saturating-exponential-plus-linear function. The standard error associated with each individual De determination was estimated by Monte Carlo simulation. Curve fitting, De determination and Monte Carlo

simulation were performed using version 3.24 of the Luminescence Analyst software[30]. A selection of OSL decay and growth curves are presented in Supplementary Fig. 7.

It has been observed widely that the majority of quartz grains from unheated sedimentary deposits do not yield a measureable OSL signal[31-34]. Similarly, a large number of grains display luminescence characteristics which indicate that they are unsuitable for age determination. Consequently, single-grain dating studies must adopt criteria for rejecting uninformative grains[32]. In this study grains were rejected where one or more of the following conditions are met: (1) the natural signal from the grain is too low to distinguish it from the variability in the background signal (the difference between Tn and the average count from the background interval is less than three times the standard deviation of the counts in the background interval, determined using the "sig. >3 sigma above BG" rejection criterion in Luminescence Analyst,[30]); (2) the recycling ratio[22] differs from unity by greater than 10 %; (3) the IR-depletion ratio[35] is greater than two standard errors below unity; (4) recuperation is high (i.e. Lx/Tx for the 0 Gy regeneration point is greater than 5 % of Ln/Tn,[22]); (5) the De is greater than twice the curve fitting parameter D0 when the growth curve is fitted using a single saturating exponential saturating exponential fit[36,37] i.e. the grain has received a natural radiation dose which is sufficiently close to the saturation dose to preclude the calculation of an accurate De value, termed "signal saturation" hereafter, and (6) the dose response curve shape precludes the generation of a meaningful De. These rejection criteria were applied to grains in the order listed above and only one cause for rejection was noted per grain. The results of this analysis are presented in Supplementary Table 3. Of the 16,500 grains measured, only 1,026 displayed acceptable luminescence characteristics, a yield of 6.2 %. However, there is considerable inter-sample variability in yield, with yields from individual samples ranging from 1.9 % to 13.7 %. Comparable values of 1.7 % and 12.7 % were obtained for samples from Blombos Cave, South Africa[32,38]. The majority of grains were rejected due to either low natural sensitivity (15 %), poor recycling ratio (57 %) or signal saturation (14 %).

Rejection rates due to the signal saturation criterion are strongly dose dependant with ~7.5 % of grains being rejected at lower doses (OSL13: 47 Gy and OSL11: 59 Gy) and ~19.5 % of grains being rejected at higher doses (OSL5 and 3, both ~110 Gy). The use of D0 as the basis of a rejection criterion is not universal in single-grain OSL dating. However, recent research[39,40] has demonstrated that the application of a rejection criterion which excludes grains with low D0 values can yield a significant improvement in dose recovery ratios. Thomsen et al.[40] showed that for a known dose of 107 Gy, their sample yields dose recovery ratios which are consistent with unity where $D0 \geq De$, but below unity where $D0 < De$. Using our less stringent rejection criterion ($2*D0 \geq De$), all samples yield dose recovery ratios consistent with unity, indicating that accurate "natural" De values should be obtained using this value. Application of this less stringent criterion has the advantage of increasing the yield of acceptable grains.

For HF acid etched sand-sized quartz grains, the environmental dose rate consists of external beta, gamma and cosmic ray components. An alpha dose rate due to uranium and thorium within quartz grains has been included in several recent studies[41], but is here omitted. Beta and gamma dose rates may be calculated from the water content and radioisotope concentration of the host sediments, while cosmic dose rates are calculated from the sample location (latitude, longitude and latitude) and burial depth. Radioisotope concentrations of the host sediments were measured using ICP-MS (U and Th) and ICP-AES (K) and are presented in Supplementary Table 4. These measurements were performed on dried, homogenised sub-samples of the light-exposed material removed from the sample tube prior to extraction of the dating sample. Dose rates were

calculated using the conversion factors of ref. [42], assuming that the present day radioisotope concentrations had prevailed throughout the burial period. Secular equilibrium in the uranium and thorium decay series was assumed. Dose rates were corrected for the effects of HF etching[43], grain size[44] and measured present-day water content of the sample. Present-day water contents were assigned a 3% uncertainty, which encompasses the full range of likely burial conditions. Cosmic ray dose rates were calculated using site location (4°S, 39°E, 160 m elevation) and present day sediment burial depths, assuming a sediment overburden density of 1.85 g/cm3[45] and a 6m limestone overburden with a density of 2.6 g/cm3. Because PYS13 is located in a narrow, steep-sided gorge, the dose due to cosmic rays from directly above the site, and which do not pass through the overlying limestone, is assumed to be negligible. Dose rates are presented in Supplementary Table 4.

Supplementary Table 3. The number of single grains which were measured, rejected after application of the criteria outlined in Supplementary Note 2, and accepted for inclusion in the calculation of the burial dose.

Layer	9	11	13	15	16	17	18
Sample (PYS13-OSL...)	13	11	9	17	7	5	3
Total number of grains measured							
	2300	2400	2400	2200	2400	2400	2400
Grains rejected for the following reasons							
T_n signal <3*background	459	417	263	291	215	496	361
Poor recycling ratio	1146	1344	1439	1213	1852	1202	1262
Depletion by IR	19	16	14	19	0	8	18
0 Gy dose >5% of L_n/T_n	102	110	146	127	41	159	162
Signal saturation	195	164	339	398	238	416	520
Poor DR curve	64	110	26	31	9	37	26
Sum of rejected grains							
	1985	2161	2227	2079	2355	2318	2349
Acceptable individual D_e values							
	315	239	173	121	45	82	51

To determine the age of a sample which has been measured using single-grain OSL techniques, it is first necessary to analyze the distribution of individual De values in order to generate a single burial dose (Db). Hereafter, Db is used to signify the dose estimate which is appropriate for use in age calculation. In the first instance, Db was calculated for all samples using the central age model (CAM). The CAM[46] generates Db on the assumption that the dispersion of measured De values is explained by the overdispersion parameter (σd, the relative standard deviation of the true paleodoses) and measurement uncertainties[46]. Consequently, it is appropriate for samples where the luminescence signal of all constituent grains was fully bleached prior to deposition, and where no post-depositional mixing has occurred.

Supplementary Table 4. Sample depths, water content and dose rate for PYS13.

Layer	Sample name (PYS13-…)	Sample depth (m)	Moisture Content (%)	Dose rate (Gy/ka)			Total dose rate, D_r (Gy/ka)
				Beta	Gamma	Cosmic	
9	OSL13	1.0±0.2	4.6±3	0.49±0.04	0.47±0.03	0.068±0.006	1.02±0.05
11	OSL11	1.4±0.2	8.5±3	0.60±0.05	0.54±0.04	0.066±0.006	1.21±0.06
13	OSL9	1.8±0.2	6.7±3	0.57±0.04	0.54±0.04	0.063±0.007	1.17±0.06
15	OSL17	2.0±0.2	6.5±3	0.63±0.05	0.60±0.04	0.062±0.006	1.30±0.06

16	OSL7	2.0±0.2	3.5±3	0.76±0.06	0.58±0.04	0.062±0.006	1.41±0.07
17	OSL5	2.5±0.2	7.0±3	0.73±0.05	0.75±0.05	0.059±0.007	1.53±0.07
18	OSL3	2.6±0.2	6.3±3	0.75±0.06	0.65±0.04	0.059±0.007	1.44±0.07

Equivalent dose distributions for each sample are shown in Supplementary Fig. 8, while CAM Des and overdispersion values are given in Supplemenary Table 3. The overdispersion values for the samples are all higher than 20%, which has frequently been used as a threshold above which incomplete bleaching prior to deposition and/or post-depositional mixing may be inferred (e.g. ref. [25]). However, recently overdispersion values of up to 28% for fired hearth rocks where partial bleaching and post-depositional mixing are impossible have been reported[47], and there is an increasing body of literature suggesting that overdispersion values of up to ~35% may occur in well bleached, undisturbed sediments[48,49]. In addition, the equivalent dose distributions presented in Supplementary Fig. 8 do not suggest that multiple populations of ages are present in our samples. Consequently, Db and ages were initially calculated using the CAM for all samples (Supplementary Table 5).

Supplementary Table 5. Summary dating results and ages. Uncertainties in the age estimates are based on the propagation, in quadrature, of errors associated with individual errors for all measured quantities. In addition to uncertainties calculated from counting statistics, errors due to (1) beta source calibration (3 %); (2) single-grain instrument reproducibility (1.5 %); (3) dose rate conversion factors (3 %) and attenuation factors (3 %) have been included[50]. Age estimates in parentheses are regard as unreliable and discounted from our age model for the excavation.

Layer	Sample (PYS13-…)	D_b calculation method (n)	D_b (Gy)	σd (%)	Total dose rate, D_r (Gy/ka)	Age (ka)
9	OSL13	CAM (315)	47.0±0.9	30±1	1.02±0.05	45.9±2.7
11	OSL11	CAM (239)	59.3±1.3	32±2	1.21±0.06	49.0±3.0
13	OSL9	CAM (173)	75.1±2.1	34±2	1.17±0.06	64.2±4.1
15	OSL17	CAM (121)	77.7±2.7	34±3	1.30±0.06	59.9±4.0
		CAM (45)	103±4	26±4		(72.9±5.4)
16	OSL7	FMM-1 (10)	79.2±18.5	20	1.41±0.07	(56.2±13.5)
		FMM-2 (35)	111±10	20		(78.9±8.4)
17	OSL5	CAM (82)	115±4	29±3	1.53±0.07	74.9±5.1
18	OSL3	CAM (51)	107±4	24±3	1.44±0.07	73.8±5.2

The CAM ages for all samples are in stratigraphic order, with no statistically significant age inversions. However, sedimentology (Supplementary Note 2) indicates a lithologic boundary between Layers 16 and 17, yet the CAM age for sample OSL7 (Layer 16) is consistent with that for the underlying layers 17 and 18. Although the overdispersion value for this samples (26±4%) does not suggest the presence of multiple age populations, it is possible that material from both Layers 16 and 17 was sampled when OSL7 was collected. To test this hypothesis, the finite mixture model[51] was applied to sample OSL7, following the method outlined by Jacobs et al[52]. This model treats log De values as a random sample from a mixture of normal populations with identical overdispersion[53], and may be used to determine the De of two or more populations in a mixture. This analysis provided circumstantial evidence supporting the hypothesis that OSL7 contains a mixture of material from the younger Layer 16, and older Layer 17, since the younger De component (FMM-1 in Supplementary Table 5) is consistent with that for the overlying Layer 15 (79.2±18.5 and 77.7±2.7 Gy respectively) while the older De component (FMM-2 in Supplementary Table 5) is consistent with that for the underlying Layer 17 (111±10 and 115±4 Gy respectively). However, this analysis does not provide unequivocal evidence that sample OSL7 is a mixture since (1) analysis of a dataset using the method outlined above will return a minimum of two De components irrespective of the nature of the De distribution analysed and (2) the younger De component (FMM-1) only contains 10 grains and the De is very imprecisely known. No more quartz is available from sample OSL7. Based upon the discrepancy between the CAM age and site accumulation history inferred from soil micromorphology, and upon the tentative evidence for mixing provided by the FMM analysis above, we regard the CAM and FMM ages for OSL7 as unreliable and discount them from our

age model for the excavation. CAM ages for all other samples presented in Supplementary Table 5 are regarded as reliable. The datum for these ages is 2015.

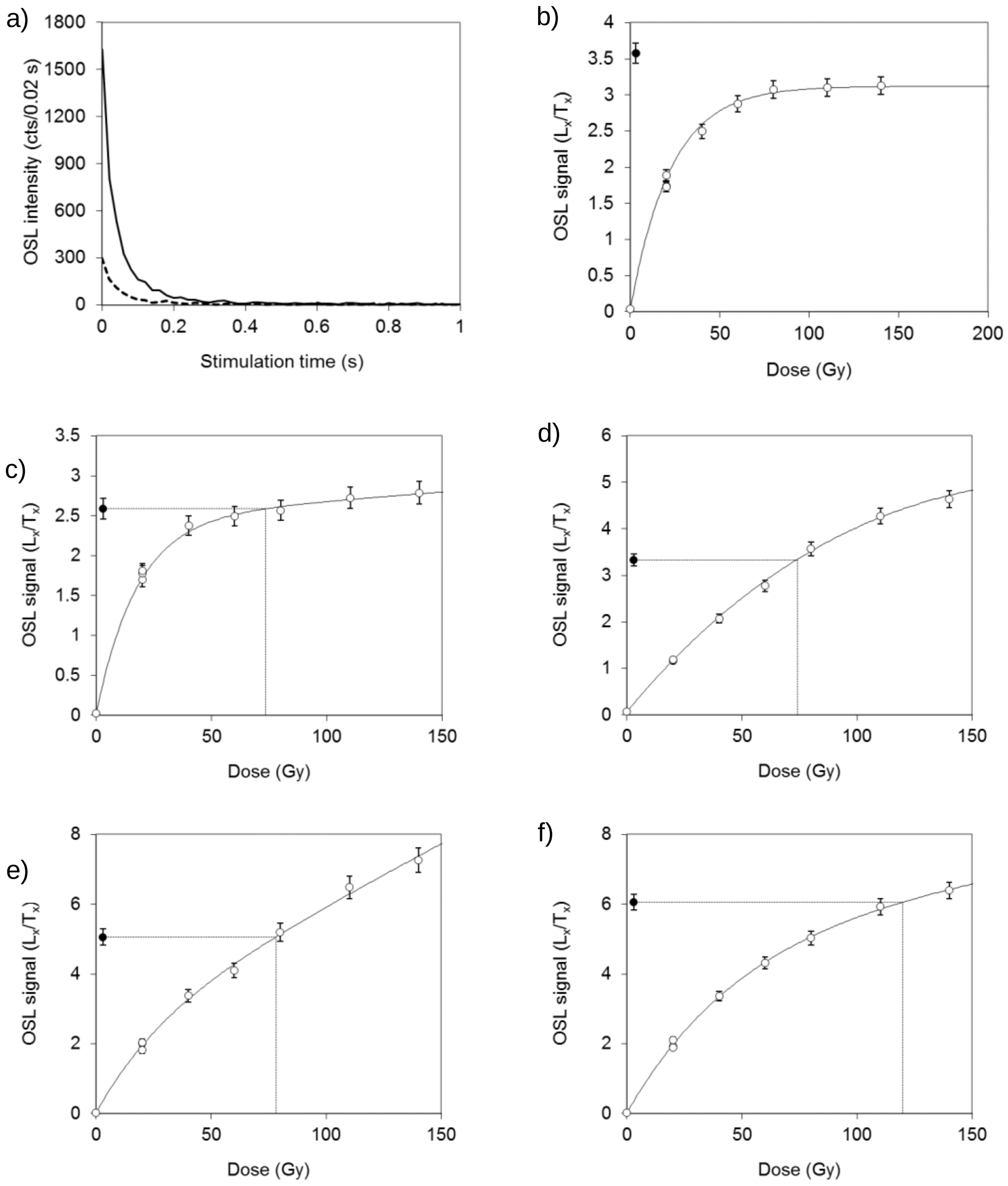

Supplementary Figure 7. Typical OSL decay and growth curves for individual grains of sample OSL17 (Layer 15). (**a**) Natural and test dose OSL decay curves for the growth curve displayed in panel **d**. (**b**) a grain for which L_n/T_n does not intercept the growth curve. (**c**) A grain where L_n/T_n intercepts the growth curve at the point where growth has effectively ceased, precluding the calculation of a meaningful D_e. (**d**, e) Grains yielding D_e values close to the mode. (**f**) A grain yielding a large D_e. Panels **b-f**: L_n/T_n (closed circle), L_x/T_x (open circles).

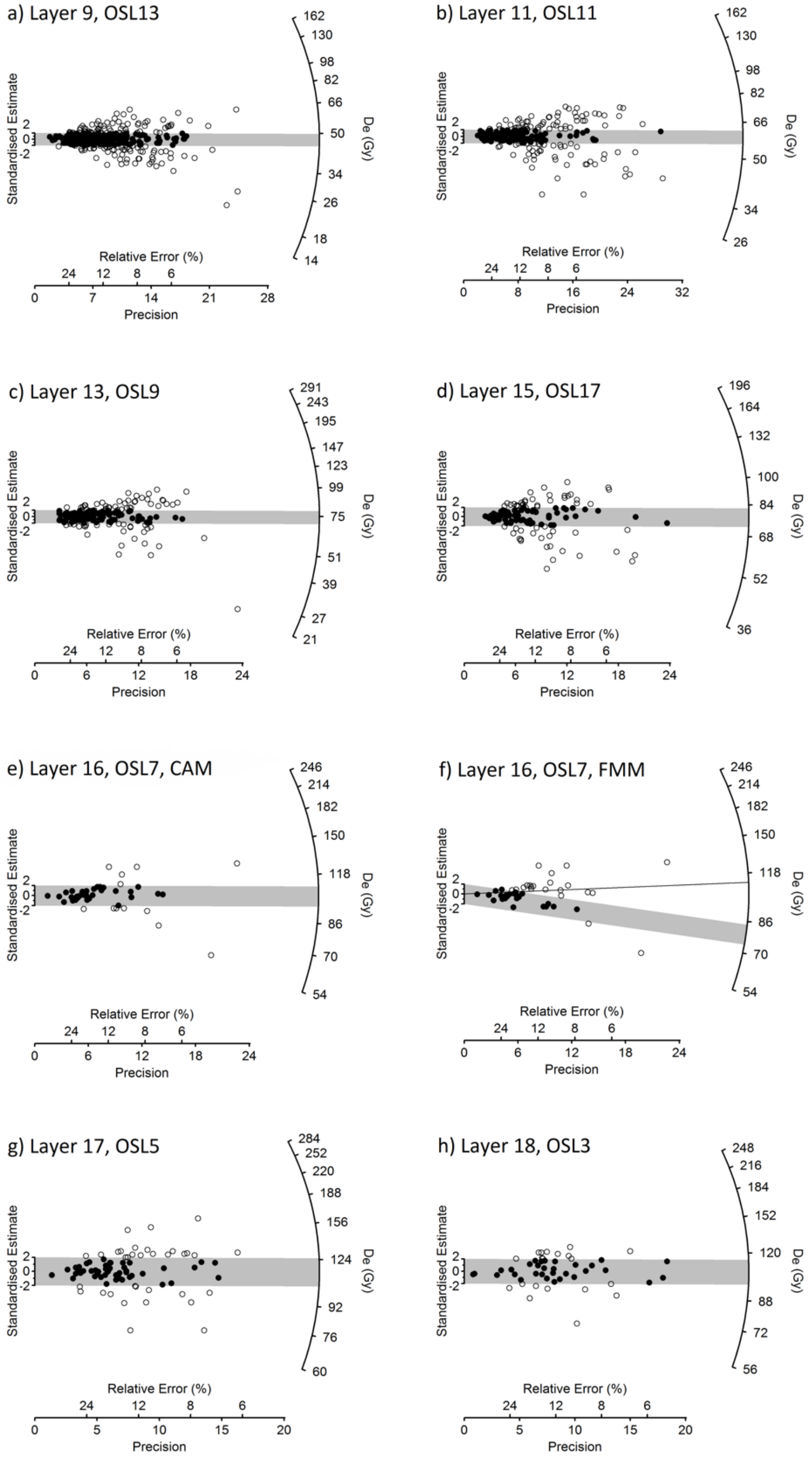

Supplementary Figure 8. Radial plots of the single grain dose distributions for PYS samples. The grey bar is centred on D_b determined using the Central Age Model, except for panel **f**, where the grey bar represents De for component FMM-1 and the black line represents De for component FMM-2, both of which were determined using the Finite Mixture Model (see Supplementary Note 2).

Bayesian Modelling

Bayesian modelling enables the relative stratigraphic information recorded during excavation to be formally incorporated into age estimates along with the calibrated probability distributions. Bayes' theorem, which encapsulates the mathematical basis of the Bayesian method, incorporates three principal components, which allow the analysis of the relationship between data (represented by y) and unknown values, or parameters, denoted by θ.

Bayes' theorem is:

$$p(\theta|y) \propto p(y|\theta)\, p(\theta)$$

Here, $p(y|\theta)$ is defined as the 'likelihood', where p is a probability function (the symbol | means 'given'), so the likelihood tells us how likely are the values of the data we observed, given some specific values of the unknown parameters. Calibrated radiocarbon and OSL probability distributions represent the likelihood. $p(\theta)$ denotes the 'prior'. This term describes our strength of belief concerning the possible values of unknown parameters before, or prior to, the observation of the data we collect. $p(\theta|y)$, the 'posterior', is a probability function that reflects the level of confidence associated with the values of the unknown parameters after the observation of the data. Bayes' theorem therefore may be written as; 'the posterior belief is proportional to the likelihood times the prior'. In terms of archeological dating, this can be understood as meaning that 'the modelling results are proportional to the probability derived from the radiocarbon and OSL dates multiplied by the probabilities defined by constraints of the law of superimposition in stratigraphy'.

Supplementary Fig. 9 shows the results of the Bayesian model, Supplementary Table 6 shows the age ranges from which the ages in Fig. 1B were taken, and supplementary Table 7 shows the estimated time interval represented by each Layer.

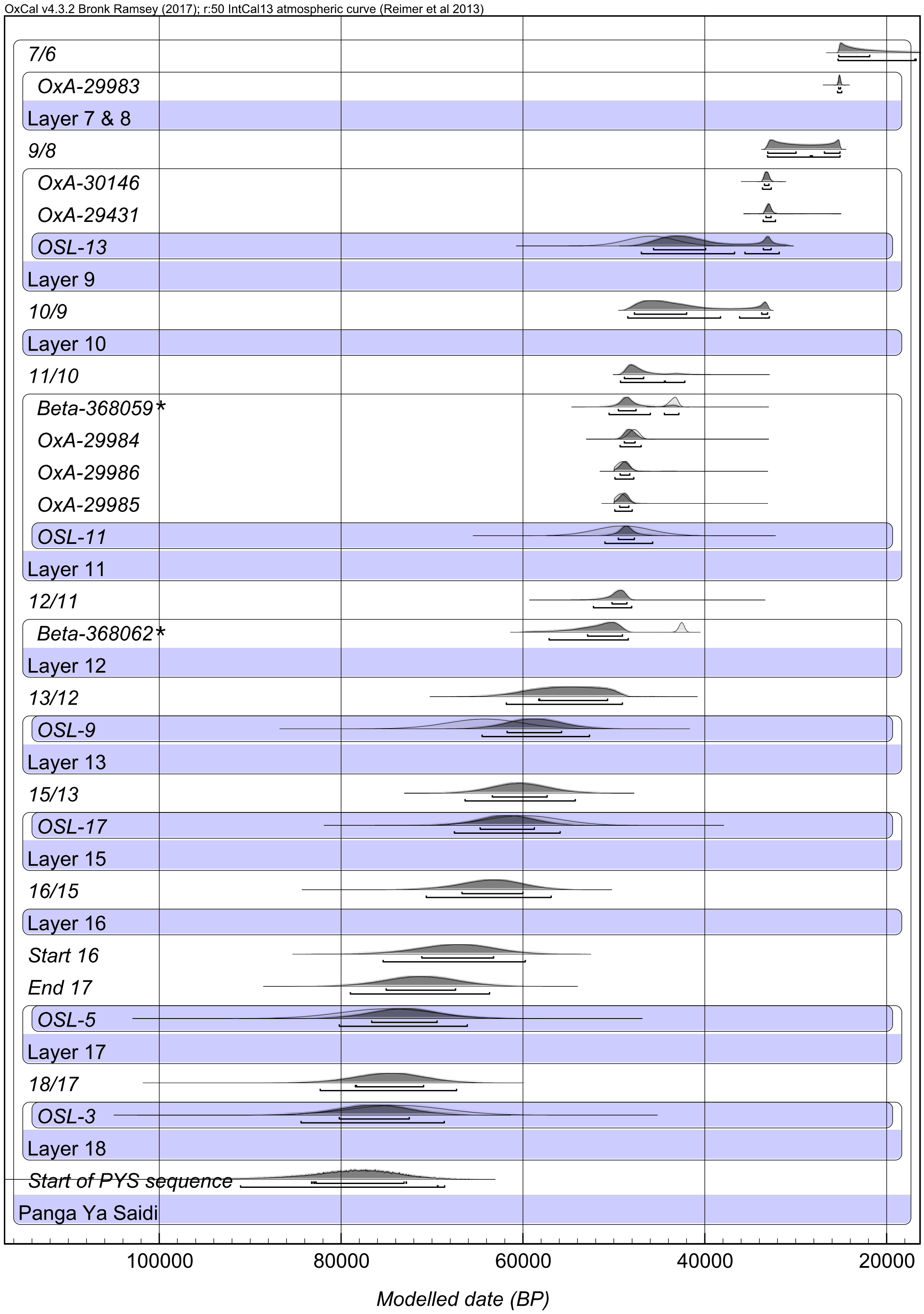

Supplementary Figure 9. Bayesian model of all available determinations from Layers 18 to 7 from PYS produced using OxCal v. 4.3. OSL-7 was deemed unreliable and was omitted due to reasons explained in the text above. The model identifies the 2 Beta radiocarbon dates as 100% outliers (marked with *) and excludes them. Produced using OxCal v4.3.2[54] and IntCal13[55].

Supplementary Table 6. Date ranges following the application of a Bayesian model including all available determinations from Layers 18 to 7 from PYS produced using OxCal v. 4.3. OSL-7 was deemed unreliable and was omitted due to reasons explained in the text above. The model identifies the 2 Beta radiocarbon dates as 100% outliers (marked with *) and excludes them.

MODEL	**Calibrated/ Unmodelled (BP)**				**Calibrated/ Modelled (BP)**			
	from	to	from	to	from	to	from	to
	68.20%		95.40%		68.20%		95.40%	
End 7/Start 6					*25300*	*21850*	*25350*	*16750*
OxA-29983	25300	25050	25400	24900	25300	25050	25400	24900
**Layer 7 & 8**								
End 9/Start 8					*33100*	*25100*	*33100*	*25100*
OxA-30146	33450	32950	33650	32700	33450	32900	33650	32650
OxA-29431	33250	32650	33500	32350	33300	32700	33600	32200
OSL-13	48700	43100	51300	40500	45650	32650	47000	31800
**Layer 9**								
End 10/Start 9					47750	33050	48500	32900
**Layer 10**								
End 11/Start 10					*48850*	*46700*	*49300*	*42150*
Beta-368059	43850	42900	44400	42600	49500	47550	50550	42800
OxA-29984	48350	47150	49000	46700	48850	47650	49350	47000
OxA-29986	49650	48400	...	47900	49300	48250	49900	47800
OxA-29985	49750	48650	...	48150	49400	48350	49900	47950
OSL-11	52100	45900	55000	43000	49550	47700	51000	45700
**Layer 11**								
End 12/Start 11					*50250*	*48550*	*52250*	*48000*
Beta-368062	42850	42200	43200	41950	52900	49050	57150	48400
**Layer 12**								
End 13/ start 12					58300	50650	61850	49050
OSL-9	68400	60000	72400	56000	61750	55750	64500	52650
**Layer 13**								
End 15/Start 13					*63400*	*57300*	*66400*	*54200*
OSL-17	64000	55800	67900	51900	64700	58700	67550	55900
**Layer 15**								
End 16/Start 15					*66700*	*60000*	*70650*	*56850*
**Layer 16**								
Start 16					*71150*	*63200*	*75400*	*59700*
End 17					75050	67400	79000	63650
OSL-5	80150	69650	85100	64700	76650	69400	80250	66100
**Layer 17**								
End 18/Start 17					*78450*	*70900*	*82300*	*67300*
OSL-3	79150	68450	84200	63400	80200	72500	84450	68650
**Layer 18**								
Start of 18 /Start of PYS sequence					*83250*	*72800*	*91100*	*68600*

Supplementary Table 7. Estimated periods of duration for Layers 18 to 7 based on the application of a Bayesian model including all available determinations produced using OxCal v. 4.3.

DURATIONS	**Calibrated- Modelled (BP)**			
	From	to	from	to
	68.20%		95.40%	
Layers 7-8	28700	23500	32150	20500
Layer 9	40800	31050	45400	27450
Layer 10	48450	43850	48950	36850
Layer 11	49550	47700	51200	44500
Layer 12	53850	49100	58300	48500
Layer 13	61250	54500	64000	51200
Layer 15	65000	58750	68300	55600
Layer 16	68900	61650	72900	58350
Layer 17	76600	69150	80450	65500
Layer 18	80700	72150	86350	67950

Supplementary Note 4: Full details of the results of cultural artifact analyses

Lithics

Complete elongate flakes with their technological length more than twice as long as their width were classified here as blades (*sensu lato*). Blades do not exceed 8% of the debitage frequency for any given layer at PYS (Supplementary Fig. 10), though there is some evidence for the application of organized blade production strategies at various stages in the sequence. Blade frequency starts out low (<2.5% of all debitage) in Layers 17-19 where there is no evidence for the application of systematic strategies to create blades as end products. Blade frequency slightly increases in Layers 15-16, with some single platform and bipolar blade production, but then decreases somewhat between Layers 9 and 14 (Supplementary Fig. 10). From Layer 8 upwards blade production is more frequent, and in these upper layers blades were produced through the application of a diversity of strategies including bipolar, Levallois, and prismatic blade core reduction (Supplementary Figs. 11-13).

Evidence for Levallois core reduction also has a flickering presence through the sequence. Levallois flakes were identified in Layers 18 and 19, Layers 10-14, as well as in Layers 3-5 (Supplementary Figs. 10, 11, 13, and 14; Supplementary Data 2). However the strategies employed to prepare Levallois flaking-surfaces varied among these layers. The Levallois products from Layers 18 and 19 were relatively large (35-99 mm in technological length), with recurrent unidirectional and centripetal preparation (Supplementary Fig. 12; Supplementary Data 3). In Layers 10-14 recurrent centripetal Levallois technology was employed and the cores and products were relatively small (31-43 mm in length) (Supplementary Fig. 14). In Layers 3-5 both recurrent unidirectional and bidirectional Levallois strategies (Supplementary Fig. 14) were employed to produce small elongate flakes (Supplementary Fig. 11). Levallois technology is rare in some parts of Africa in the Holocene, but is also reported in an assemblage from the site of Goda Buticha in Ethiopia[56].

Bipolar cores were abundant through much of the PYS sequence, but were absent in Layers 19-17 (Supplementary Figs. 10, 12, and 13). Low frequencies of bipolar flakes did occur in these lower layers however, indicating that bipolar strategies were practiced. Similarly, although no bipolar cores were recovered from Layer 10 there were occasional bipolar flakes in this layer indicating that the technology did not disappear completely at any stage within the sequence. Supplementary Fig. 13 shows an abrupt increase in the frequency of bipolar cores from Layers 11 to 16 (~67-48.5ka), paralleling the increase in bipolar in the early LSA deposits in the Eastern Rift Valley at Mumba ~57ka[57], as well as at Olduvai Gorge and Ntuka River[58], and Border Cave in southern Africa by ~43ka[59].

Backed crescents were the most abundant type of retouched artifact, and occurred in two parts of the sequence, namely, in Layers 11 and 12 and in Layers 2-6 (Fig. 3; Supplementary Figs. 10 and 16). Their presence in layers dating to ~48.5ka accords with findings from younger LSA sites elsewhere in eastern Africa such as Mochena Borago and Enkapune ya Muto[60,61].

The three major knapped raw materials at PYS occur throughout the sequence: limestone, quartz, and chert (Fig. 2; Supplementary Fig. 15). Sources of all three occur within a 10 km radius of the cave. The shift observed in rock type frequencies between Layers 16 and 17 is abrupt (Fig. 2; Supplementary Fig. 15). To confirm the significance of this change a chi-square test was conducted on raw material frequencies of flakes and flaked pieces (angular fragments) in Layer 17, versus those in Layers 15 and 16 (which were lumped to increase sample size:

Supplementary Table 8), showing a significant reduction in the proportion of limestone ($P<0.001$).

Artifact size also decreases abruptly across the transition (Fig. 2; Supplementary Figs. 17 and 18). To investigate this decrease in artifact size, an unequal variances t-test was conducted on artifact weight for all flakes and flaked pieces in Layer 17, versus those in Layers 15 and 16 (Supplementary Table 9). One possibility is that this decrease in artifact size is a function of the shift in raw material usage across the transition. In other words different raw materials were available in different package sizes, thereby dictating the maximum size of artifacts producible on them. However, even when t-tests are conducted within fine-grained raw materials (quartz and chert) there is still a significant reduction in artifact size between Layer 17 and Layers 15 and 16 (Supplementary Table 10). Furthermore this decrease in artifact size is also evident in retouched artifacts (Supplementary Table 11).

As Figure 3 indicates, not only are these changes in rock type frequency and debitage size abrupt, they characterize the entire remainder of the sequence. This is in contrast to bipolar technology which occurs throughout the sequence albeit in varying frequencies, and it is in contrast to Levallois and blade technology which occur only intermittently (Supplementary Figs. 10 and 13). The presence of backing also does not provide a consistent signature for the emergence of the LSA, as it appears in Layers 11 and 12 but then is absent after this point until the relatively recent Layer 6 (<25ka) (Supplementary Figs. 10 and 16). Despite flickering changes in technology that occur through the sequence, there is a marked and consistent shift towards the production of small fine-grained stone flakes from 67,000 years ago onwards. Therefore we suggest this transition marks the onset of the LSA at PYS.

Supplementary Table 8. The distribution of raw materials either side of the major occupational hiatus at the top of Layer 17. A chi-squared test showed this difference to be significant at the $P<0.00001$ level (test statistic = 237.7065).

Rock Type	Layers 15 & 16	Layer 17
Chert	38	26
Quartz	224	13
Limestone	21	115

Supplementary Table 9. T-test comparing weight in grams of flakes and flaked pieces.

Layer	N	Mean	Std. Deviation	Std. Error Mean	t	df	Sig.
15 & 16	281	1.59	2.63	.16	-4.451	158.725	<0.001
17	153	6.34	13.06	1.06			

Supplementary Table 10. Unequal variances t-test comparing the weight in grams of quartz and chert flakes and flaked pieces between Layers 15-16 and 17. (Note that sample size of limestone in Layers 15 and 16 was too small to be able to conduct the test for this material.)

Layer	N	Mean	Std. Deviation	Std. Error Mean	T	df	Sig.
15 & 16	258	1.12	1.18	0.07	-3.148	37.698	0.003
17	38	3.52	4.67	0.76			

Supplementary Table 11. Mean retouched weight in grams.

Layers	Mean Weight	N
2 to 3	1.554	23
4	2.215	30
5 to 6	4.293	27
9 to 13	8.454	47
17 to 19	22.103	12

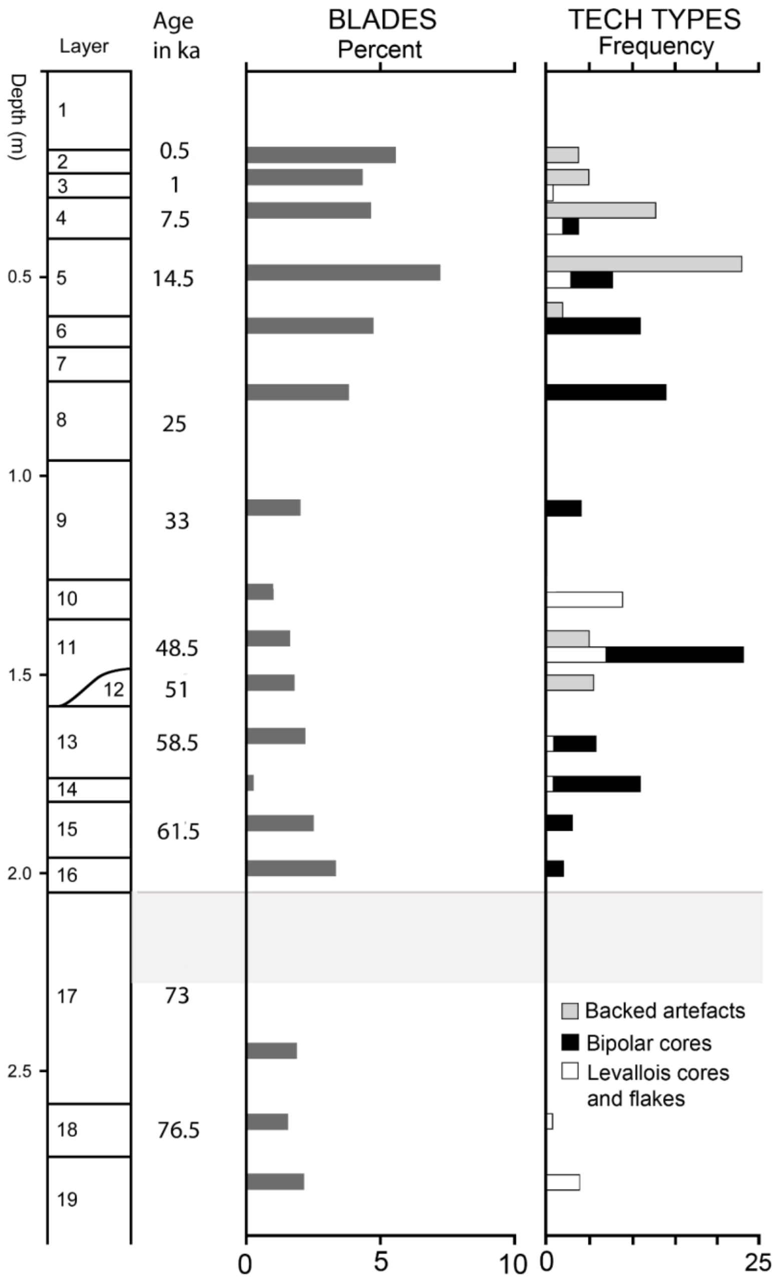

Supplementary Figure 10. Overview of key diagnostic lithic types in the PYS sequence in relation to stratigraphy and ages.

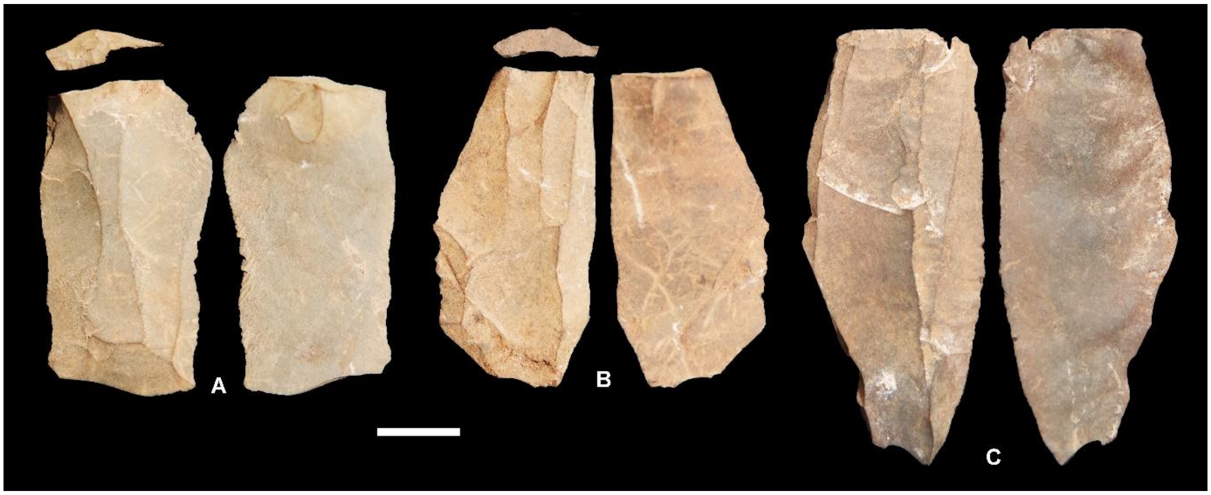

Supplementary Figure 11. Chert blades from Panga ya Saidi. A and B = Layer 4, C = Layer 6. A has a facetted platform and is likely from a Levallois core, B has overhang removal and is likely from a prismatic core. The scale is 1cm.

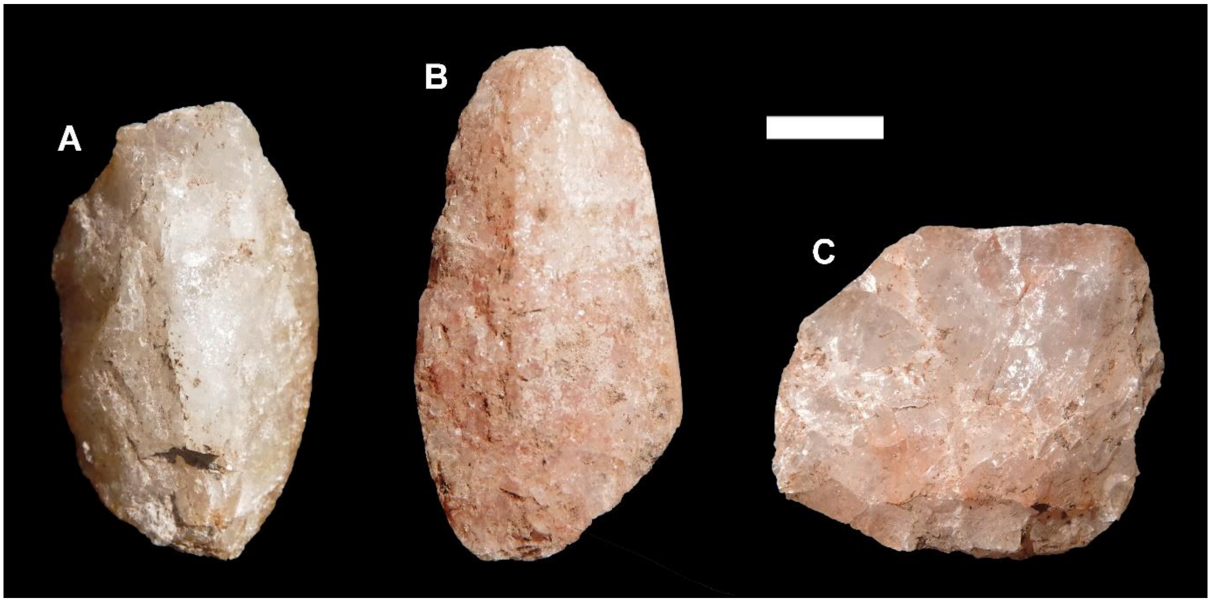

Supplementary Figure 12. Bipolar quartz cores from Layer 8 at Panga ya Saidi. Note that pieces A and B are flaked on their long axis and would have produced blades. The piece on the right (C) has been rotated and flaked on both axes. The scale is 1cm.

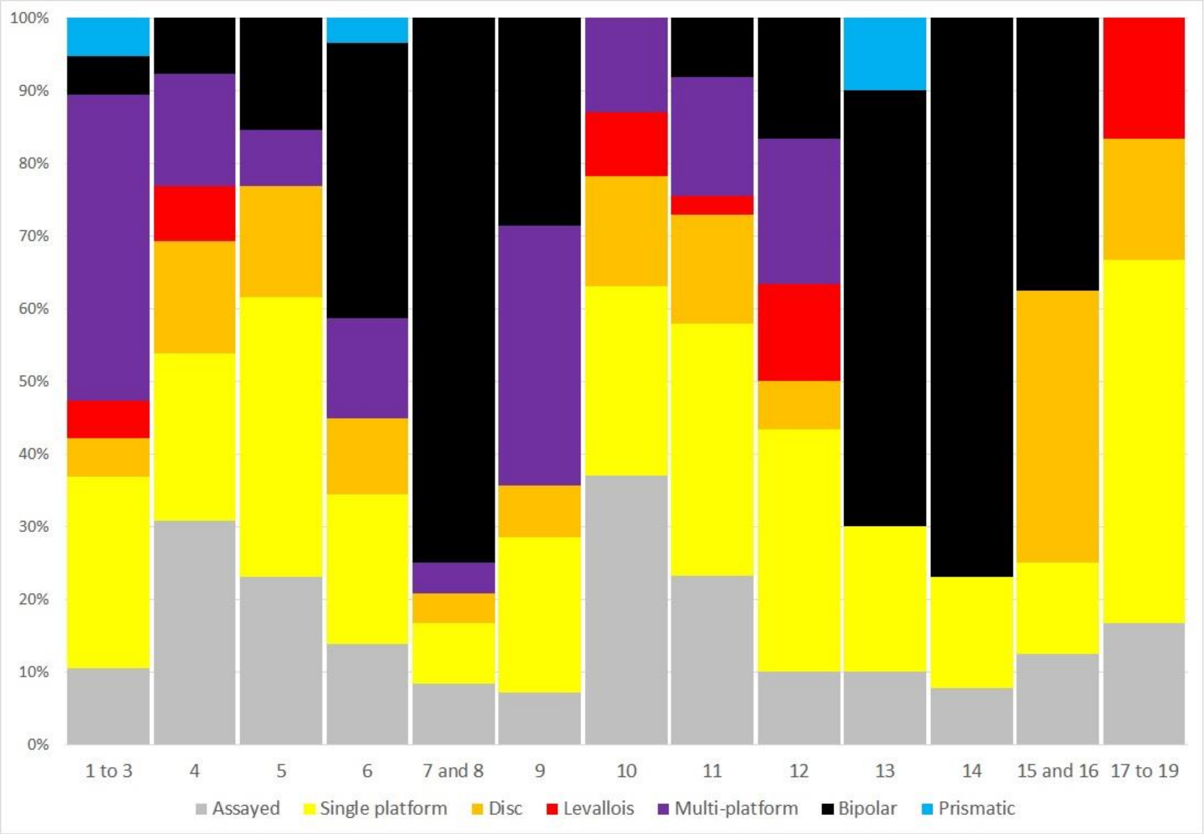

Supplementary Figure 13. Proportion of different core types in the layers of the PYS sequence. Assayed cores are those with less than three scars. Disc cores include discoidal and hierarchical discs.

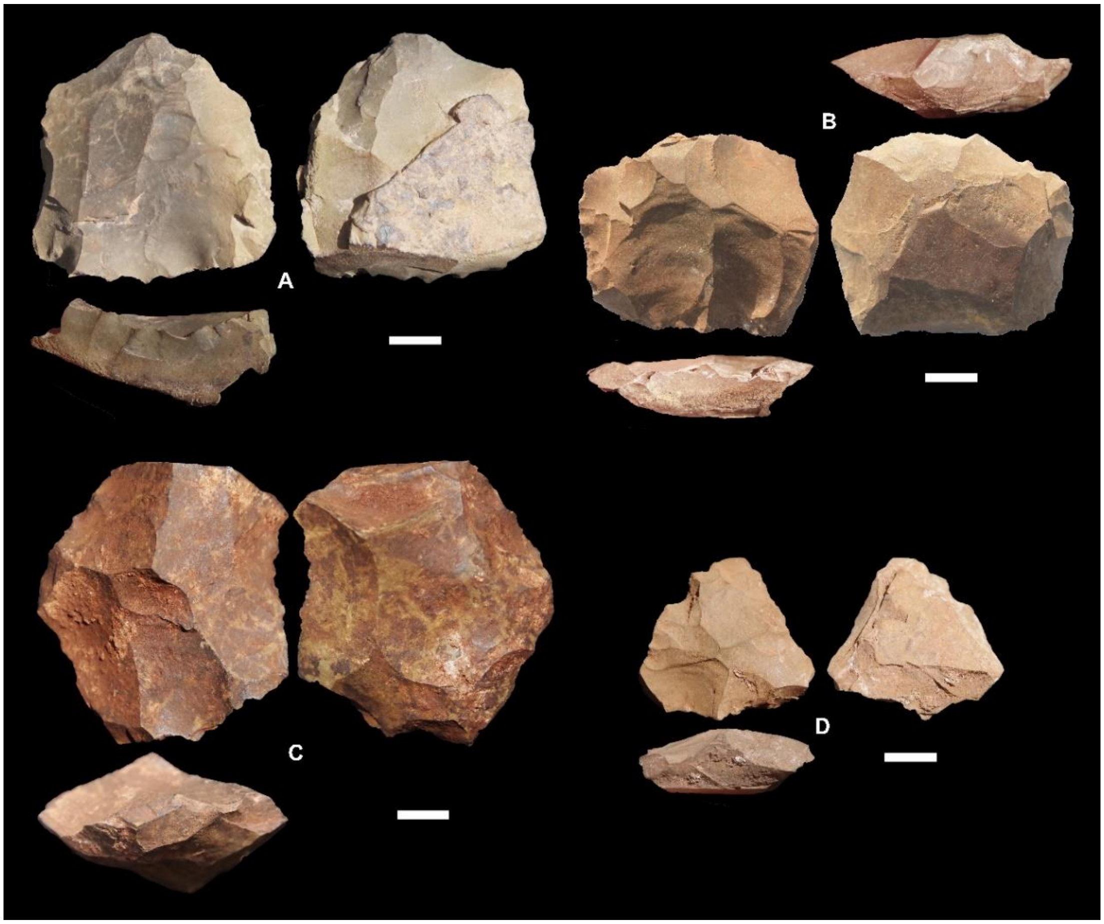

Supplementary Figure 14. Chert Levallois cores from Panga ya Saidi. A = Layer 5, B = Layer 3, C = Layer 19, D = Layer 11. The scales are 1cm.

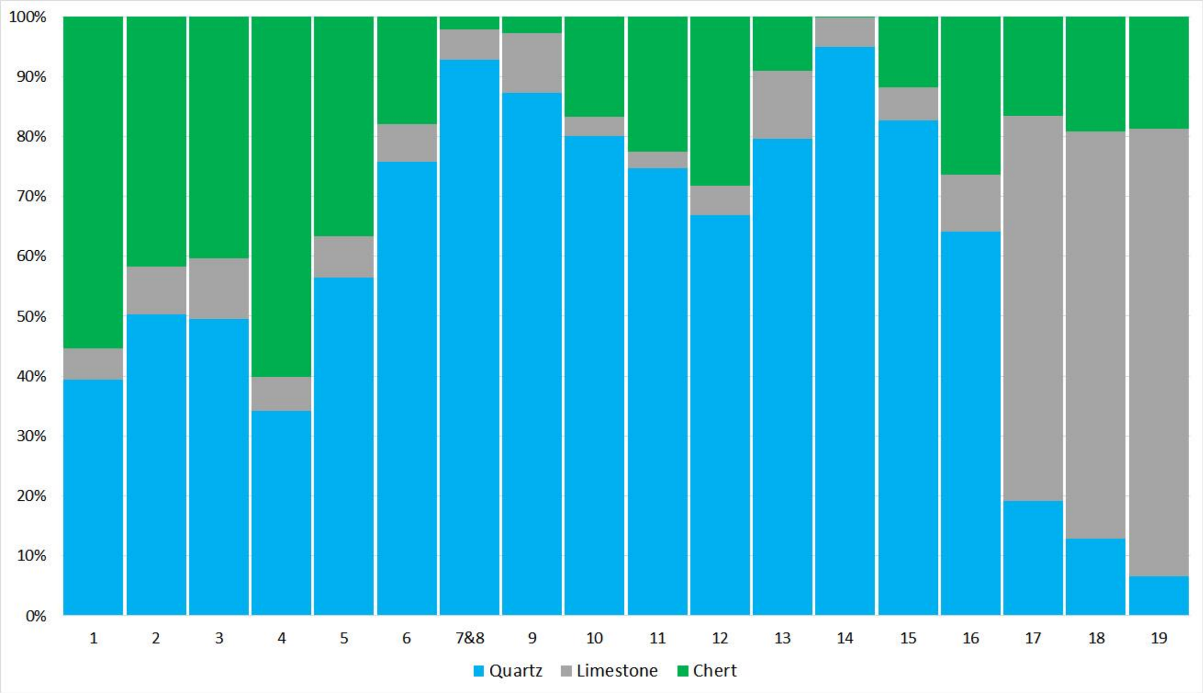

Supplelementary Figure 15. Proportion of raw material types by artifact count and Layer at Panga ya Saidi.

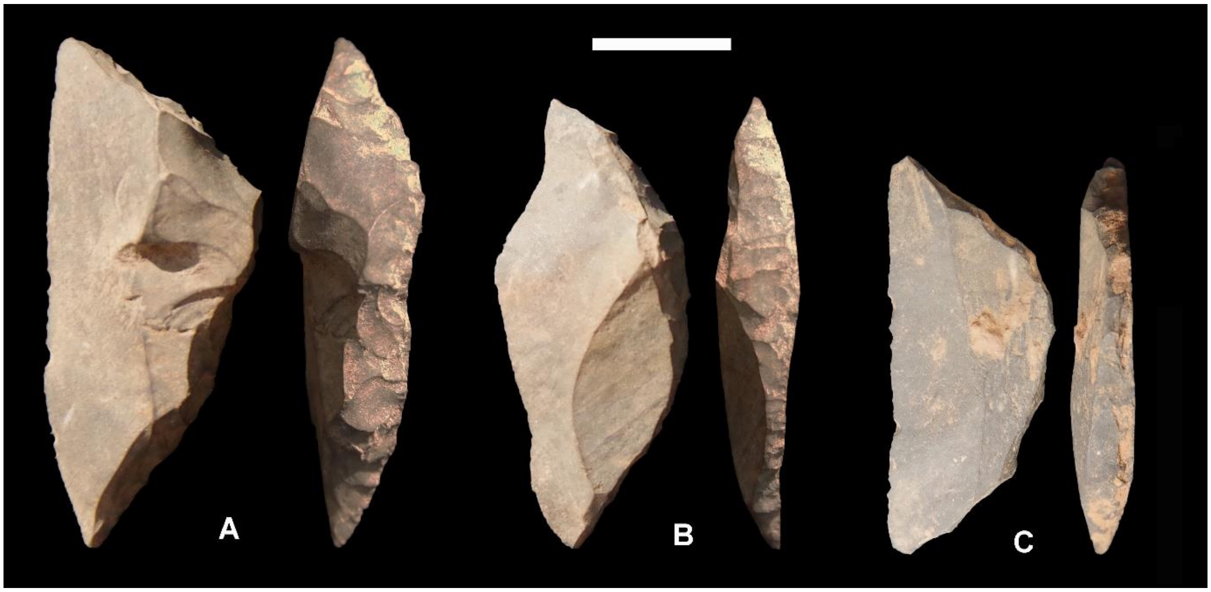

Supplementary Figure 16. Chert crescents from Panga ya Saidi. A and B = Layer 5, C = Layer 12. The scale is 1cm.

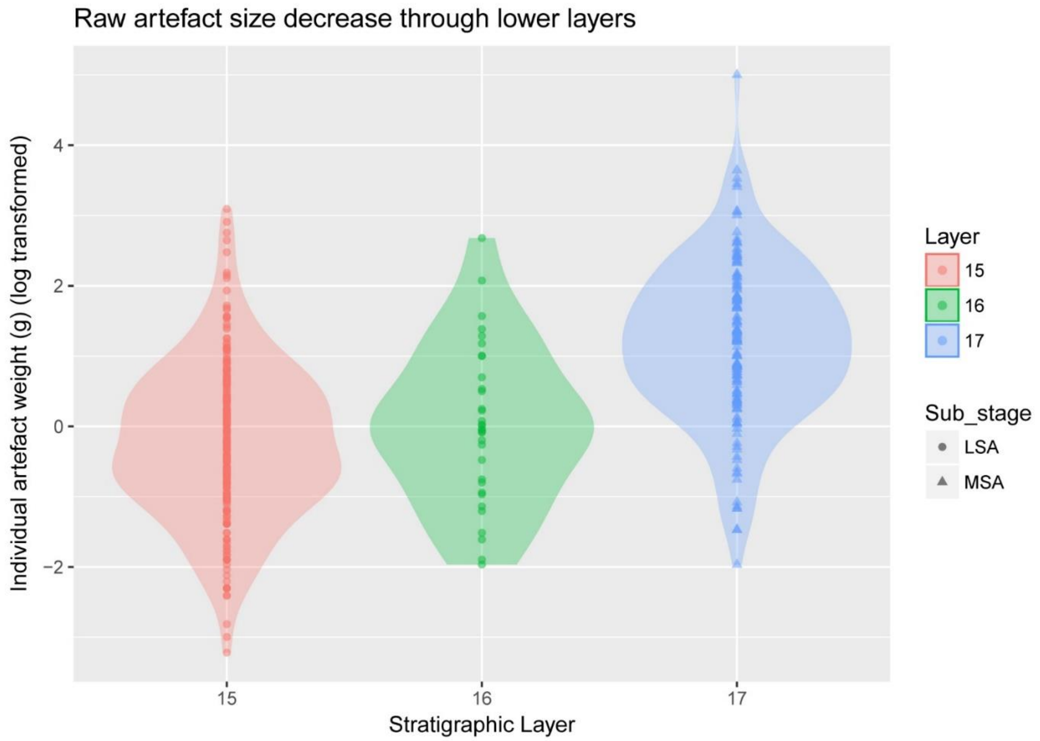

Supplementary Figure 17. Violin plots of the size of flakes and flaked pieces in Layers 15, 16, and 17.

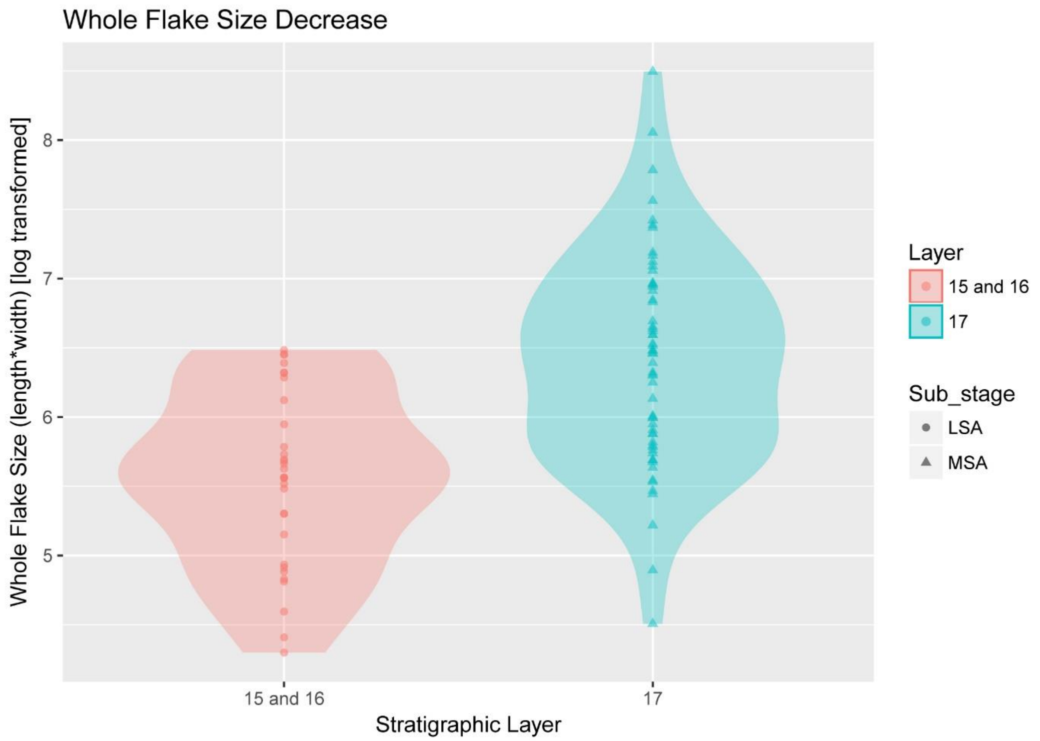

Supplementary Figure 18. Violin plot of the size of whole flakes (length*width), for Layers 15 and 16 combined and Layer 17.

Beads, osseous artifacts, and ochre

Taphonomic analysis of *Conus* shells was based on Claassen[62], Mattes[63], Harding[64] and d'Errico and Backwell[65]. The identification of traces of manufacture, heating and use wear on beads made of marine shells and ostrich egg shells relied on diagnostic criteria established experimentally[66-68] and previous analyses of similar objects[69-71]. The distinction between natural and anthropogenic modifications on worked bone was based on criteria defined in the literature and the analysis of modern and Pleistocene reference collections[72-76]. Identification of shaping techniques and use-wear was based on data from experimental bone tool manufacture and use[75,77-79], and experimental reproduction and microscopic analysis of marks produced with different tools and motions[80]. The identification of grinding, scraping and traces of deliberate engraving on ochre fragment is based on criteria obtained from experimental research[81-84], examinations of engravings on stone and bone surfaces[80], and by comparison with published engraved pieces from Blombos Cave and Klasies River[71,85,86].

The distribution of beads, osseuous artifacts, and ochre through the PYS sequence is shown in Supplementary Fig. 19 where it can be seen there is a gradual increase between 61.5 and 25 ka, followed by a particular combination of artefacts characterizing the Holocene.

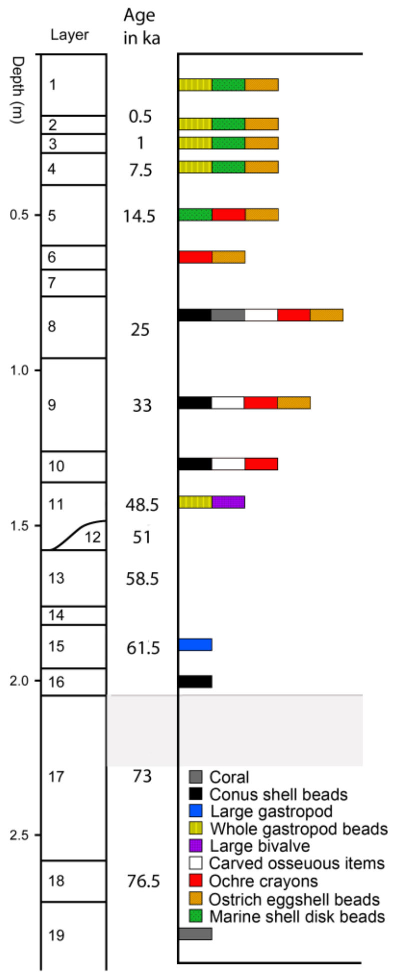

Supplementary Figure 19. The presence of beads (n=223), osseuous artifacts (n=7), ochre crayons (n=5), and marine manuports (n=4) in the layers of the PYS sequence.

Supplementary Note 5: Full details of the results of palaeoecological analyses

Phytoliths

Our key types include globular granulates (Supplementary Fig. 20n) and the blocky type (Supplementary Fig. 20m) from dicot trees and shrubs[87-89]. Very large blocky phytoliths (Supplementary Fig. 20r-t) have been reported for the trees and shrubs of the Clusiaceae[87]. Tabular sinuates derive from woody sources[88] (Supplementary Fig. 20z, ac). Some arboreal types are diagnostic at the family level such as the tabular oblong dendritic for the Burseraceae (Supplementary Fig. 20k), and the tabular thick dendritic (Supplementary Fig. 20e) for the Bignoniaceae[87]. Epidermal polygonal assemblages (Supplementary Fig. 20b) derive from leaf tissue, and their cell shape and wall outline and thickness denote affiliation with the Euphorbiaceae[87]. Palms produce globular echinates[90] (Supplementary Fig. 20v-w). The Poaceae are represented by long saddles (Supplementary Fig. 20o), which derive from the wet-adapted bambusoids[91], 'towers' (Supplementary Fig. 20c) are from xeric chloridoid grasses[91]. Lobates (Supplementary Fig. 20d come from tall, hydric, heliophyte panicoid grasses[91]. Lastly, globular, 'ridged' types with echinoid tendencies (Supplementary Fig. 20ab) derive from the Mararantaceae[92], a forest adapted family.

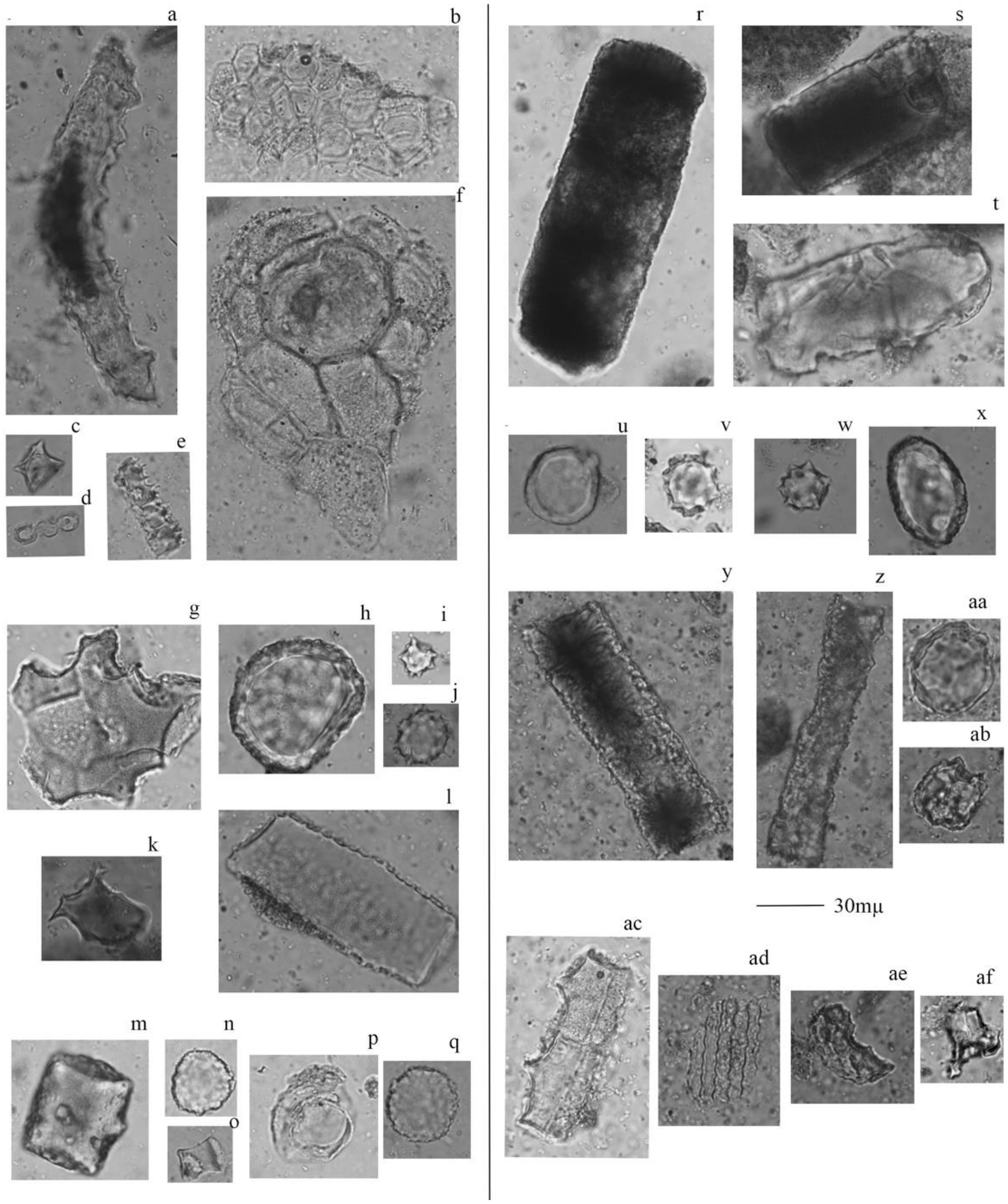

Supplementary Figure 20. Phytolith morphotypes and provenance-depth. (**a**) Large tabular oblong radiating (8-10cm); (**b**) Epidermal polygonal (22-24cm); (**c**) Tower (22-24 cm); (**d**) Polylobate (22-24 cm); (**e**) Tabular thick dendritic (22-24 cm); (**f**) Hair base (22-24 cm); (**g**) Large blocky facetate (64-66cm); (**h**) Large globular granulate (64-66cm); (**i**) Globular echinate (48-50cm); (**j**) Large globular echinate (48-50cm); (**k**) Tabular oblong dendritic (48-50cm); (**l**) Large blocky psilate (64-66cm); (**m**) Blocky (82-84cm); (**n**) Globular granulate (82-84cm); (**o**) Long saddle (82-84cm); (**p**) Large hemisphere, psilate (82-84cm); (**q**) Large globular granulate (82-84cm); (**r**) Large blocky (112-114cm); (**s**) Large blocky (112-114cm); (**t**) Large blocky (112-114cm); (**u**) Large hemisphere, psilate (abaxial) (146-148cm); (**v**) Globular echinate (146-148cm); (**w**) Globular echinate (146-148cm); (**x**) Large ovate granulate (146-148cm); (**y**) Large tabular thick (164-166cm); (**z**) Large tabular thick (164-166cm); (**aa**) Large globular granulate (164-166cm); (**ab**) Globular ridged (164-166cm); (**ac**) Tabular thick sinuate (174-176cm); (**ad**) Epidermal long cells (174-176cm); (**ae**) Globular facetate, side view (174-176cm); (**af**) Sclereid (174-176cm).

Terrestrial Mollusks

The preservation of the shells is remarkable, and many extremely small species are well represented in the samples. Throughout, the fauna is typical of that of modern coastal forest habitats in East Africa, including those at Arabuko-Sokoke and on the Shimba Hills[93,94]. Most of the species represented at PYS are extant and endemic to the coastal strip (Zanzibar-Inhambane mosaic); the only notable absences are the Veronicellidae (which do not possess a shell and are therefore unlikely to enter the fossil record) and species considered to be modern introductions to the region. At least 71 morphospecies have so far been recorded, an extremely high number for a coastal site; the richest modern forest faunas average around 30 species. Some of this diversity may be the result of turnover between samples. However, rates of mollusk species endemism mean that high turnover between ecologically similar forests is a feature of the coastal fauna[95]. It has not yet been possible to identify some taxa precisely, primarily because many aspects of the distribution and ecology of the African molluskan fauna remain unknown, but some species from the early part of the PYS sequence appear to be at least locally extinct. *Truncatellina* cf. *obesa*, for example, has not previously been recorded near the coast, having a central African known distribution. The presence of this species suggests that there was once more continuous forest cover across low latitude Africa, but recent fragmentation of this habitat has led to its extirpation in East Africa.

In general, the habitat surrounding PYS appears to have been remarkably stable. Terrestrial mollusks are only diverse and abundant in East Africa today in heavily-vegetated habitats, desiccation and insolation being major causes of mortality. As sample size of individual specimens varies between layers, a rarefaction analysis was conducted (Supplementary Fig. 21) before assessing the mollusk species diversity shown in Fig. 2 and Supplementary Fig. 22. The richest snail communities are found in forest leaf litter in shade, especially on limestone substrata as here. The seven species that occur in all of the samples include some said to occur in bushland or scrub, but which also occur in forest[96]; at the present day it would seem extremely unlikely that all seven would be found together in a poorly-vegetated habitat. It is therefore probable that the area was wooded or forested to a greater or lesser extent throughout the period studied. None of the samples records a significant reduction in diversity and abundance that would be expected in the event of a major vegetation clearance (e.g. by fire), unless the mollusk community was able to recovered rapidly enough for this to be undetectable. However, given the very small size of most of the species, the extent of habitat around PYS could still be rather small, perhaps under 100m^2. Snails are able to persist in small areas of suitable habitat for many more generations than more mobile animals. An example, albeit on a larger scale, is the genus *Gonospira*, which has never been collected outside this small area of coastal Kenya in recent times, yet occurred across Kenya in Miocene times.

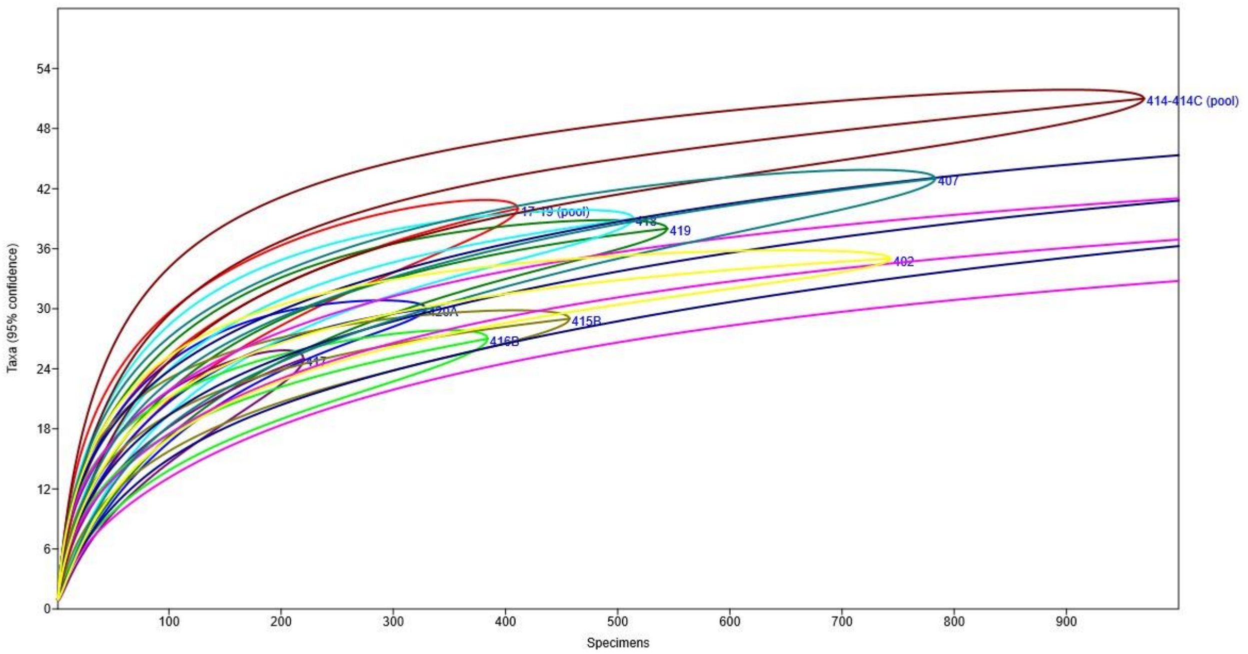

Supplementary Figure 21. Expected species richness (and 95% confidence limits) following rarefaction using PAST. Context 414-414C pool = Layer 10; 17-19 pool = Layers 17-19; 407 = Layer 2; 418 = Layer 14; 419 = Layer 15; 402 = Layer 1; 420A = Layer 16; 415B = Layer 11; 417 = Layer 13; 416D = 12. The smallest sample was context 417 (220 specimens) and also had the lowest species richness (25). The largest sample was context 413A (5668 specimens, 50 species). Y-axis truncated at 1000 specimens for clarity.

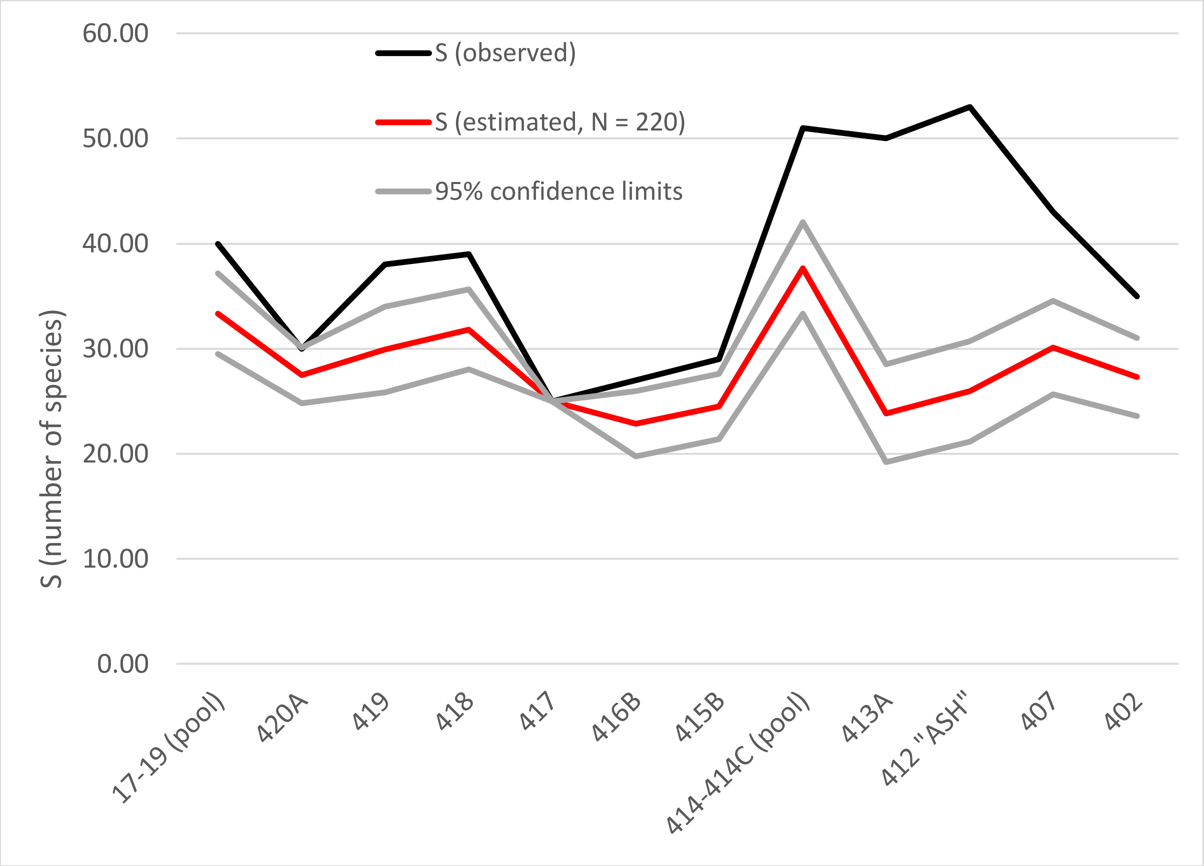

Supplementary Figure 22. Expected species richness (and 95% confidence limits) following individual rarefaction to the smallest sample size of N = 220 (sample 417, 25 species). When rarified to 220 individuals, the largest sample (413A, 23.8 species) is actually slightly less diverse than the smallest (417). The effect is to smooth the curve by depressing the peaks, especially in the later part of the sequence, although peaks for samples 17-19 (33.3 species) and 414-414C (37.7 species) remain evident. Moreover the fauna remains relatively rich (23-38 species) throughout, such high values being typical of coastal forest faunas.

<u>Tetrapod Remains</u>

The consistent presence of bush adapted species such as suni, duikers and dik diks indicates the continual presence of woodland (Fig. 2). However warthog is present in every phase from Layer 16 onwards, with exception of Layer 13, indicating PYS was never far from open country. As a measure of human occupation intensity the (MNI) proportion of bovids and suids (taxa that were likely to have entered the cave as human prey) versus bats and rodents (taxa that were unlikely to have entered the cave as human prey) were plotted in Fig. 2.

Stable Isotopes

Supplementary Table 12. Results of ANOVA for $\delta^{13}C$ by PYS Layer groupings

	**Degrees of freedom**	**Sum of suqares**	**Mean square**	**F value**	**Pr(>F)***
Layer group	5	667.2	133.44	5.723	0.000
Residuals	108	2519.1	23.32		

*p=<0.05

Supplementary Table 13. Results of post-Hoc Tukey pairwise comparison for $\delta^{13}C$ by PYS Layer groupings. 95% confidence interval of difference is indicated alongside 95% probability of lower and upper bounds of this difference.

**Layer groupings**	**Difference**	**Lower**	**Upper**	**P-value***
10 to 12-1 to 3	4.577	0.349	8.805	0.026
13 to 16-1 to 3	0.189	-4.846	5.224	1.000
17-1 to 3	-5.238	-12.416	1.941	0.286
4 to 6-1 to 3	1.704	-2.659	6.067	0.867
7 to 9-1 to 3	4.055	-1.072	9.183	0.205
13 to 16-10 to 12	-4.388	-8.711	-0.064	0.045
17-10 to 12	-9.814	-16.513	-3.116	0.001
4 to 6-10 to 12	-2.873	-6.391	0.645	0.176
7 to 9-10 to 12	-0.521	-4.952	3.909	0.999
17-13 to 16	-5.427	-12.662	1.808	0.257
4 to 6-13 to 16	1.515	-2.941	5.971	0.921
7 to 9-13 to 16	3.866	-1.340	9.073	0.268
4 to 6-17	6.941	0.157	13.726	0.042
7 to 9-17	9.293	1.994	16.592	0.005
7 to 9-4 to 6	2.351	-2.208	6.911	0.667

*p=<0.05

Supplementary Table 14. Results of ANOVA for $\delta^{18}O$ by PYS Layer groupings

	**Degrees of freedom**	**Sum of suqares**	**Mean square**	**F value**	**Pr(>F)***
Layer group	5	66.7	13.339	5.873	0.000
Residuals	108	245.3	2.271		

*p=<0.05

Supplementary Table 15. Results of post-Hoc Tukey pairwise comparison for $\delta^{18}O$ by PYS Layer groupings. 95% confidence interval of difference is indicated alongside 95% probability of lower and upper bounds of this difference.

Layer groupings	Difference	Lower	Upper	P-value*
10 to 12-1 to 3	2.063	0.743	3.382	0.000
13 to 16-1 to 3	0.867	-0.705	2.438	0.600
17-1 to 3	0.060	-2.180	2.300	1.000
4 to 6-1 to 3	0.583	-0.779	1.945	0.815
7 to 9-1 to 3	1.371	-0.229	2.972	0.137
13 to 16-10 to 12	-1.196	-2.546	0.153	0.113
17-10 to 12	-2.003	-4.093	0.088	0.069
4 to 6-10 to 12	-1.480	-2.578	-0.382	0.002
7 to 9-10 to 12	-0.691	-2.074	0.691	0.696
17-13 to 16	-0.807	-3.065	1.451	0.905
4 to 6-13 to 16	-0.284	-1.675	1.107	0.991
7 to 9-13 to 16	0.505	-1.120	2.130	0.945
4 to 6-17	0.523	-1.595	2.640	0.980
7 to 9-17	1.311	-0.967	3.590	0.554
7 to 9-4 to 6	0.789	-0.634	2.212	0.595

*p=<0.05

Supplementary References

1 Caswell, P. *Geology of the Kilifi-Mazeras area*. (Geological Survey of Kenya, 1956).
2 Thompson, A. *Geology of the Malindi area*. (Geological Survey of Kenya, 1956).
3 Abuodha, J. Geomorphological evolution of the southern coastal zone of Kenya. *Journal of African Earth Sciences* **39**, 517-525 (2004).
4 Horkel, A. *et al.* Notes on the geology and mineral resources of the southern Kenyan coast. *Mitteilungen der österreichische geologische Gesellschaft* **77**, S151-S159 (1984).
5 Oosterom, A. P. *The geomorphology of southeast Kenya*, Landbouwuniversiteit te Wageningen, (1988).
6 Moomaw, J. *Plant Ecology of the Coast Region of Kenya Colony, British East Africa*. (Kenya Department of Agriculture, 1960).
7 Robertson, S. & Luke, W. *Kenya coastal forests: The report of the NMK/WWF coastal forest survey. Kenya coast forest status, conservation and management*. (Nairobi: National Museums of Kenya, 1993).
8 Sombroek, W. G., Braun, H. & Van der Pouw, B. *Exploratory soil map and agro-climatic zone map of Kenya, 1980. Scale 1: 1,000,000*. (Kenya Soil Survey, 1982).
9 Otto-Bliesner, B. L., Marshall, S. J., Overpeck, J. T., Miller, G. H. & Hu, A. Simulating Arctic climate warmth and icefield retreat in the last interglaciation. *Science* **311**, 1751-1753 (2006).
10 Braconnot, P. *et al.* Results of PMIP2 coupled simulations of the Mid-Holocene and Last Glacial Maximum–Part 2: feedbacks with emphasis on the location of the ITCZ and mid-and high latitudes heat budget. *Climate of the Past* **3**, 279-296 (2007).
11 Hijmans, R. J., Cameron, S. E., Parra, J. L., Jones, P. G. & Jarvis, A. Very high resolution interpolated climate surfaces for global land areas. *International Journal of Climatology* **25**, 1965-1978 (2005).
12 Viles, H. Blue-green algae and terrestrial limestone weathering on Aldabra Atoll: An SEM and light microscope study. *Earth Surface Processes and Landforms* **12**, 319-330 (1987).
13 Viles, H., Spencer, T., Teleki, K. & Cox, C. Observations on 16 years of microfloral recolonization data from limestone surfaces, Aldabra Atoll, Indian Ocean: implications for biological weathering. *Earth Surface Processes and Landforms* **25**, 1355-1370 (2000).
14 Herries, A. I. Archaeomagnetic evidence for climate change at Sibudu Cave. *Southern African Humanities* **18**, 131-147 (2006).
15 Herries, A. I. & Fisher, E. C. Multidimensional GIS modeling of magnetic mineralogy as a proxy for fire use and spatial patterning: Evidence from the Middle Stone Age bearing sea cave of Pinnacle Point 13B (Western Cape, South Africa). *Journal of Human Evolution* **59**, 306-320 (2010).
16 Liu, Z., Liu, Q., Torrent, J., Barrón, V. & Hu, P. Testing the magnetic proxy χ FD/HIRM for quantifying paleoprecipitation in modern soil profiles from Shaanxi Province, China. *Global and Planetary Change* **110**, 368-378 (2013).
17 Dearing, J. A. *et al.* Frequency-dependent susceptibility measurements of environmental materials. *Geophysical Journal International* **124**, 228-240 (1996).
18 Eyre, J. K. Frequency dependence of magnetic susceptibility for populations of single-domain grains. *Geophysical Journal International* **129**, 209-211 (1997).
19 Worm, H.-U. On the superparamagnetic—stable single domain transition for magnetite, and frequency dependence of susceptibility. *Geophysical Journal International* **133**, 201-206 (1998).
20 Worm, H. U. Time-dependent IRM: A new technique for magnetic granulometry. *Geophysical Research Letters* **26**, 2557-2560 (1999).
21 Liu, Q. *et al.* An integrated study of the grain-size-dependent magnetic mineralogy of the Chinese loess/paleosol and its environmental significance. *Journal of Geophysical Research: Solid Earth* **108**, EPM7 1-14 (2003).
22 Murray, A. S. & Wintle, A. G. Luminescence dating of quartz using an improved single-aliquot regenerative-dose protocol. *Radiation Measurements* **32**, 57-73 (2000).
23 Armitage, S., Duller, G. & Wintle, A. Quartz from southern Africa: sensitivity changes as a result of thermal pretreatment. *Radiation Measurements* **32**, 571-577 (2000).
24 Armitage, S. *et al.* The formation and evolution of the barrier islands of Inhaca and Bazaruto, Mozambique. *Geomorphology* **82**, 295-308 (2006).
25 Jacobs, Z. *et al.* Ages for the Middle Stone Age of southern Africa: implications for human behavior and dispersal. *Science* **322**, 733-735 (2008).

26 Roberts, R., Galbraith, R., Olley, J., Yoshida, H. & Laslett, G. Optical dating of single and multiple grains of quartz from Jinmium rock shelter, northern Australia: Part II, results and implications. *Archaeometry* **41**, 365-395 (1999).

27 Wallinga, J., Murray, A. & Duller, G. Underestimation of equivalent dose in single-aliquot optical dating of feldspars caused by preheating. *Radiation Measurements* **32**, 691-695 (2000).

28 Jacobs, Z., Duller, G. A. & Wintle, A. G. Interpretation of single grain De distributions and calculation of De. *Radiation Measurements* **41**, 264-277 (2006).

29 Thomsen, K. J., Murray, A. & Bøtter-Jensen, L. Sources of variability in OSL dose measurements using single grains of quartz. *Radiation Measurements* **39**, 47-61 (2005).

30 Duller, G. Assessing the error on equivalent dose estimates derived from single aliquot regenerative dose measurements. *Ancient TL* **25**, 15-24 (2007).

31 Duller, G., Bøtter-Jensen, L. & Murray, A. Optical dating of single sand-sized grains of quartz: sources of variability. *Radiation Measurements* **32**, 453-457 (2000).

32 Jacobs, Z., Duller, G. A. & Wintle, A. G. Optical dating of dune sand from Blombos Cave, South Africa: II—single grain data. *Journal of Human Evolution* **44**, 613-625 (2003).

33 Rodnight, H., Duller, G., Wintle, A. & Tooth, S. Assessing the reproducibility and accuracy of optical dating of fluvial deposits. *Quaternary Geochronology* **1**, 109-120 (2006).

34 Thomsen, K. J., Murray, A., Bøtter-Jensen, L. & Kinahan, J. Determination of burial dose in incompletely bleached fluvial samples using single grains of quartz. *Radiation Measurements* **42**, 370-379 (2007).

35 Duller, G. Distinguishing quartz and feldspar in single grain luminescence measurements. *Radiation measurements* **37**, 161-165 (2003).

36 Wintle, A. G. & Murray, A. S. A review of quartz optically stimulated luminescence characteristics and their relevance in single-aliquot regeneration dating protocols. *Radiation Measurements* **41**, 369-391 (2006).

37 Durcan, J. A. *Luminescence dating of sediments in Punjab, Pakistan: implications for the collapse of the Harappan Civilisation*, Aberystwyth University, (2013).

38 Jacobs, Z., Duller, G. A., Wintle, A. G. & Henshilwood, C. S. Extending the chronology of deposits at Blombos Cave, South Africa, back to 140ka using optical dating of single and multiple grains of quartz. *Journal of Human Evolution* **51**, 255-273 (2006).

39 Gliganic, L. A., Jacobs, Z. & Roberts, R. G. Luminescence characteristics and dose distributions for quartz and feldspar grains from Mumba rockshelter, Tanzania. *Archaeological and Anthropological Sciences* **4**, 115-135 (2012).

40 Thomsen, K. *et al.* Testing single-grain quartz OSL methods using sediment samples with independent age control from the Bordes-Fitte rockshelter (Roches d'Abilly site, Central France). *Quaternary Geochronology* **31**, 77-96 (2016).

41 Vandenberghe, D., De Corte, F., Buylaert, J.-P. & Kučera, J. On the internal radioactivity in quartz. *Radiation Measurements* **43**, 771-775 (2008).

42 Adamiec, G. & Aitken, M. J. Dose-rate conversion factors: update. *Ancient TL* **16**, 37-50 (1998).

43 Hong, D.-G. *Luminescence stimulated from quartz by green light: developments relevant to dating*, University of Edinburgh, (1998).

44 Mejdahl, V. Thermoluminescence dating: Beta-dose attenuation in quartz grains. *Archaeometry* **21**, 61-72 (1979).

45 Prescott, J. & Hutton, J. Cosmic ray and gamma ray dosimetry for TL and ESR. *International Journal of Radiation Applications and Instrumentation. Part D. Nuclear Tracks and Radiation Measurements* **14**, 223-227 (1988).

46 Galbraith, R. F., Roberts, R. G., Laslett, G., Yoshida, H. & Olley, J. M. Optical dating of single and multiple grains of quartz from Jinmium rock shelter, northern Australia: Part I, experimental design and statistical models. *Archaeometry* **41**, 339-364 (1999).

47 Armitage, S. & King, G. Optically stimulated luminescence dating of hearths from the Fazzan Basin, Libya: a tool for determining the timing and pattern of Holocene occupation of the Sahara. *Quaternary Geochronology* **15**, 88-97 (2013).

48 Tribolo, C., Mercier, N., Rasse, M., Soriano, S. & Huysecom, E. Kobo 1 and L'Abri aux Vaches (Mali, West Africa): Two case studies for the optical dating of bioturbated sediments. *Quaternary Geochronology* **5**, 317-323 (2010).

49 Tribolo, C. *et al.* OSL and TL dating of the Middle Stone Age sequence at Diepkloof Rock Shelter (South Africa): a clarification. *Journal of Archaeological Science* **40**, 3401-3411 (2013).

50 Murray, A. S. & Olley, J. M. Precision and accuracy in the optically stimulated luminescence dating of sedimentary quartz: a status review. *Geochronometria* **21**, 1-16 (2002).
51 Galbraith, R. & Green, P. Estimating the component ages in a finite mixture. *International Journal of Radiation Applications and Instrumentation. Part D. Nuclear Tracks and Radiation Measurements* **17**, 197-206 (1990).
52 Jacobs, Z., Wintle, A. G., Duller, G. A., Roberts, R. G. & Wadley, L. New ages for the post-Howiesons Poort, late and final Middle stone age at Sibudu, South Africa. *Journal of Archaeological Science* **35**, 1790-1807 (2008).
53 Roberts, R. G., Galbraith, R., Yoshida, H., Laslett, G. & Olley, J. M. Distinguishing dose populations in sediment mixtures: a test of single-grain optical dating procedures using mixtures of laboratory-dosed quartz. *Radiation Measurements* **32**, 459-465 (2000).
54 OxCal version 4.3.2 (2017).
55 Reimer, P. J. *et al.* IntCal13 and Marine13 radiocarbon age calibration curves 0-50,000 years cal BP. *Radiocarbon* **55**, 1869–1887 (2013).
56 Pleurdeau, D. *et al.* Cultural change or continuity in the late MSA/Early LSA of southeastern Ethiopia? The site of Goda Buticha, Dire Dawa area. *Quaternary International* **343**, 117-135 (2014).
57 Eren, M. I., Diez-Martin, F. & Dominguez-Rodrigo, M. An empirical test of the relative frequency of bipolar reduction in Beds VI, V, and III at Mumba Rockshelter, Tanzania: implications for the East African Middle to Late Stone Age transition. *Journal of Archaeological Science* **40**, 248-256 (2013).
58 Ambrose, S. H. Small Things Remembered: Origins of Early Microlithic Industries in Sub-Saharan Africa. *Archeological Papers of the American Anthropological Association* **12**, 9-29 (2002).
59 Villa, P. *et al.* Border Cave and the beginning of the Later Stone Age in South Africa. *Proceedings of the National Academy of Sciences* **109**, 13208-13213 (2012).
60 Ambrose, S. H. Chronology of the Later Stone Age and food production in East Africa. *Journal of Archaeological Science* **25**, 377-392 (1998).
61 Brandt, S. A. *et al.* Early MIS 3 occupation of Mochena Borago Rockshelter, Southwest Ethiopian Highlands: implications for Late Pleistocene archaeology, paleoenvironments and modern human dispersals. *Quaternary International* **274**, 38-54 (2012).
62 Claassen, C. *Shells.* (Cambridge University Press, 1998).
63 Mattes, P. *Puka Shell Hawaii: the story of Puka shell jewelry in the Hawaiian Islands*. (Hawaiian Puka Shell, 1974).
64 Harding, J. Conus shell disc ornaments (vibangwa) in Africa. *The Journal of the Royal Anthropological Institute of Great Britain and Ireland* **91**, 52-66 (1961).
65 d'Errico, F. & Backwell, L. Earliest evidence of personal ornaments associated with burial: the Conus shells from Border Cave. *Journal of Human Evolution* **93**, 91-108 (2016).
66 d'Errico, F., Jardón-Giner, P. & Soler-Mayor, B. *Critères à base expérimentale pour l'étude des perforations naturelles et intentionnelles sur coquillages*. (Éditions ERAUL, 1993).
67 d'Errico, F., Henshilwood, C., Vanhaeren, M. & Van Niekerk, K. Nassarius kraussianus shell beads from Blombos Cave: evidence for symbolic behaviour in the Middle Stone Age. *Journal of Human Evolution* **48**, 3-24 (2005).
68 d'Errico, F. *et al.* Additional evidence on the use of personal ornaments in the Middle Paleolithic of North Africa. *Proceedings of the National Academy of Sciences* **106**, 16051-16056 (2009).
69 Kandel, A. W. & Conard, N. J. Production sequences of ostrich eggshell beads and settlement dynamics in the Geelbek Dunes of the Western Cape, South Africa. *Journal of Archaeological Science* **32**, 1711-1721 (2005).
70 Orton, J. Later Stone Age ostrich eggshell bead manufacture in the Northern Cape, South Africa. *Journal of Archaeological Science* **35**, 1765-1775 (2008).
71 d'Errico, F. *et al.* Early evidence of San material culture represented by organic artifacts from Border Cave, South Africa. *Proceedings of the National Academy of Sciences* **109**, 13214-13219 (2012).
72 Bonnichsen, R. & Sorg, M. *Bone Modification.* (University of Maine, 1989).
73 Villa, P. & d'Errico, F. Bone and ivory points in the Lower and Middle Palaeolithic of Europe. *Journal of Human Evolution* **41**, 69-112 (2001).
74 Backwell, L. & d'Errico, F. Termite gathering by Swartkrans early hominids. *Proceedings of the National Academy of Sciences* **98**, 1358-1363 (2001).
75 Backwell, L. & d'Errico, F. in *From Early Hominids to Modern Humans* (eds Francesco d'Errico & Lucinda Backwell) 238-275 (Witwatersrand University Press, 2005).
76 Binford, L. R. *Bones: Ancient men and modern myths*. (Academic Press, 1981).

77 Newcomer, M. H. Study and replication of bone tools from Ksar Akil (Lebanon). *World Archaeology* **6**, 138-153 (1974).
78 d'Errico, F., Giacobini, G. & Puech, P.-F. An experimental study of the technology of bone-implement manufacture. *MASCA Journal* **3**, 71-74 (1984).
79 Choyke, A. M. & Bartosiewicz, L. *Crafting bone: skeletal technologies through time and space*. Vol. 937 (Archaeopress, 2001).
80 d'Errico, F. A new model and its implications for the origin of writing: the La Marche antler revisited. *Cambridge Archaeological Journal* **5**, 163-206 (1995).
81 d'Errico, F. & Nowell, A. A new look at the Berekhat Ram figurine: implications for the origins of symbolism. *Cambridge Archaeological Journal* **10**, 123-167 (2000).
82 Soressi, M. & d'Errico, F. Pigments, gravures, parures: les comportements symboliques controversés des Néandertaliens. *Les Néandertaliens. Biologie et cultures*, 297-309 (2007).
83 Hodgskiss, T. Identifying grinding, scoring and rubbing use-wear on experimental ochre pieces. *Journal of Archaeological Science* **37**, 3344-3358 (2010).
84 Rifkin, R. F. Processing ochre in the Middle Stone Age: Testing the inference of prehistoric behaviours from actualistically derived experimental data. *Journal of Anthropological Archaeology* **31**, 174-195 (2012).
85 Henshilwood, C. S. *et al.* Emergence of modern human behavior: Middle Stone Age engravings from South Africa. *Science* **295**, 1278-1280 (2002).
86 Henshilwood, C. S., d'Errico, F. & Watts, I. Engraved ochres from the middle stone age levels at Blombos Cave, South Africa. *Journal of Human Evolution* **57**, 27-47 (2009).
87 Mercader, J., Bennett, T., Esselmont, C., Simpson, S. & Walde, D. Phytoliths in woody plants from the Miombo woodlands of Mozambique. *Annals of Botany* **104**, 91-113 (2009).
88 Runge, F. The opal phytolith inventory of soils in central Africa—quantities, shapes, classification, and spectra. *Review of Palaeobotany and Palynology* **107**, 23-53 (1999).
89 Neumann, K., Fahmy, A., Lespez, L., Ballouche, A. & Huysecom, E. The Early Holocene palaeoenvironment of Ounjougou (Mali): phytoliths in a multiproxy context. *Palaeogeography, Palaeoclimatology, Palaeoecology* **276**, 87-106 (2009).
90 Albert, R. M., Bamford, M. K. & Cabanes, D. Palaeoecological significance of palms at Olduvai Gorge, Tanzania, based on phytolith remains. *Quaternary International* **193**, 41-48 (2009).
91 Mercader, J. *et al.* Poaceae phytoliths from the Niassa Rift, Mozambique. *Journal of Archaeological Science* **37**, 1953-1967 (2010).
92 de Albuquerque, E. S. B., Braga, J. M. A. & Vieira, R. C. Morphological characterisation of silica phytoliths in Neotropical Marantaceae leaves. *Plant Systematics and Evolution* **299**, 1659-1670 (2013).
93 Lange, C. N. & Mwinzi, M. Snail diversity, abundance and distribution in Arabuko Sokoke forest, Kenya. *African Journal of Ecology* **41**, 61-67 (2003).
94 Rowson, B., Warren, B. & Ngereza, C. Terrestrial molluscs of Pemba Island, Zanzibar, Tanzania, and its status as an “oceanic” island. *ZooKeys* **70**, 1-39 (2010).
95 Tattersfield, P. Patterns of diversity and endemism in East African land snails, and the implications for conservation. *Journal of Conchology* **2**, 77-86 (1998).
96 Verdcourt, B. The penial armature of three species of east African Streptaxidae (Gastropoda, Stylommatophora). *Folia Malacologica* **8**, 215-221 (2000).
