# Supplementary material for: 78,000-year-old record of Middle and Later Stone Age innovation in an East African tropical forest
Source: Nat Commun. 2018 May 9;9:1832. doi: 10.1038/s41467-018-04057-3 (PMC5943315; doi:10.1038/s41467-018-04057-3)
Supplement: Supplementary file 2 — Description of Additional Supplementary Files [file 41467_2018_4057_MOESM2_ESM.pdf]

Description of Additional Supplementary Files

File Name: Supplementary Data 1
Description: *$\delta^{13}C$ and $\delta^{18}O$ measurements of PYS faunal samples analysed in this study including Phase grouping, sample number, stratigraphic layer, and taxonomic and tooth identifications.*

File Name: Supplementary Data 2
Description: 3D model of Layer 19 Levallois flake. Model created using Geomagic®, all rights reserved.

File Name: Supplementary Data 3
Description: 3D model of Layer 10 Levallois core. Model created using Geomagic®, all rights reserved.
