# Supplementary figures and images for: 78,000-year-old record of Middle and Later Stone Age innovation in an East African tropical forest
Source: Nat Commun. 2018 May 9;9:1832. doi: 10.1038/s41467-018-04057-3 (PMC5943315; doi:10.1038/s41467-018-04057-3)

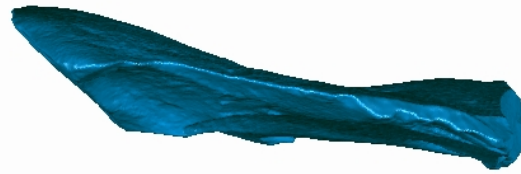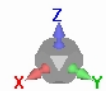

Click on the image to activate the 3D Model.

Supplement: Supplementary file 4 — Supplementary Data 2 [file 41467_2018_4057_MOESM4_ESM.pdf]

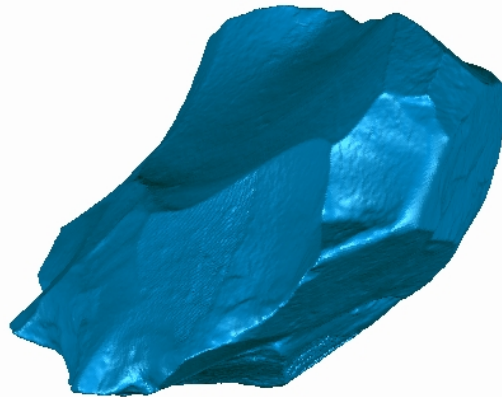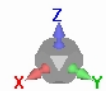

Click on the image to activate the 3D Model.

Supplement: Supplementary file 5 — Supplementary Data 3 [file 41467_2018_4057_MOESM5_ESM.pdf]
